# An exploration of mechanisms underlying *Desemzia incerta* colonization resistance to methicillin-resistant *Staphylococcus aureus* on the skin

**DOI:** 10.1128/msphere.00636-23

**Published:** 2024-02-28

**Authors:** Monica Wei, Simon A. B. Knight, Hossein Fazelinia, Lynn Spruce, Jennifer Roof, Emily Chu, Daniel Y. Kim, Preeti Bhanap, Jasmine Walsh, Laurice Flowers, Jun Zhu, Elizabeth A. Grice

**Affiliations:** 1 Department of Dermatology and Microbiology, University of Pennsylvania, Perelman School of Medicine, Philadelphia, Pennsylvania, USA; 2 Children’s Hospital of Philadelphia, Proteomics Core Facility, Philadelphia, Pennsylvania, USA; University of Nebraska Medical Center College of Medicine, Omaha, Nebraska, USA

**Keywords:** *S. aureus*, microbiome, colonization resistance, skin

## Abstract

Colonization of human skin and nares by methicillin-resistant *Staphylococcus aureus* (MRSA) leads to the community spread of MRSA. This spread is exacerbated by the transfer of MRSA between humans and livestock, particularly swine. Here, we capitalized on the shared features between human and porcine skin, including shared MRSA colonization, to study novel bacterial mediators of MRSA colonization resistance. We focused on the poorly studied bacterial species *Desemzia incerta*, which we found to exert antimicrobial activity through a secreted product and exhibited colonization resistance against MRSA in an *in vivo* murine skin model. Using parallel genomic and biochemical investigation, we discovered that *D. incerta* secretes an antimicrobial protein. Sequential protein purification and proteomics analysis identified 24 candidate inhibitory proteins, including a promising peptidoglycan hydrolase candidate. Aided by transcriptional analysis of *D. incerta* and MRSA cocultures, we found that exposure to *D. incerta* leads to decreased MRSA biofilm production. These results emphasize the value of exploring microbial communities across a spectrum of hosts, which can lead to novel therapeutic agents as well as an increased understanding of microbial competition.

**IMPORTANCE:**

Methicillin-resistant *Staphylococcus aureus* (MRSA) causes a significant healthcare burden and can be spread to the human population via livestock transmission. Members of the skin microbiome can prevent MRSA colonization via a poorly understood phenomenon known as colonization resistance. Here, we studied the colonization resistance of *S. aureus* by bacterial inhibitors previously identified from a porcine skin model. We identify a pig skin commensal, *Desemzia incerta*, that reduced MRSA colonization in a murine model. We employ a combination of genomic, proteomic, and transcriptomic analyses to explore the mechanisms of inhibition between *D. incerta* and *S. aureus*. We identify 24 candidate antimicrobial proteins secreted by *D. incerta* that could be responsible for its antimicrobial activity. We also find that exposure to *D. incerta* leads to decreased *S. aureus* biofilm formation. These findings show that the livestock transmission of MRSA can be exploited to uncover novel mechanisms of MRSA colonization resistance.

## INTRODUCTION

*Staphylococcus aureus* is a bacterial pathogen that can infect multiple body sites, including the skin, lung, heart valves, blood, and bone (1). *S. aureus* is the most commonly identified cause of skin and soft tissue infections (SSTIs) (2) and causes a significant healthcare burden (3, 4); hospitalizations related to *S. aureus* infections in the United States have an estimated annual cost of over 3 billion dollars (5). Methicillin-resistant *S. aureus* (MRSA) is particularly difficult to treat due to resistance to commonly used antibiotics. Asymptomatic colonization of skin and nares by *S. aureus* is an independent risk factor for subsequent SSTI (6, 7); colonized individuals are at a fourfold increased risk for *S. aureus* SSTI within 4 years (7). *S. aureus* spreads among individuals, especially those who are hospitalized (8) or live in the same household (9). Livestock workers are also at risk of colonization from livestock-associated MRSA strains (10). Thus, colonization not only increases the risk of later *S. aureus* skin infection but also is a source of community spread of *S. aureus*.

The commensal skin microbiota prevents colonization and invasion by pathogens such as *S. aureus*, a phenomenon known as colonization resistance (11). One mechanism of colonization resistance is the secretion of antimicrobial peptides and proteins (12). Host-produced antimicrobial proteins such as LL-37 and defensins may also reduce colonization by *S. aureus* (13, 14). Both live bacteria and their secreted products can be co-opted for therapeutic effect. For example, *Staphylococcus lugdunensis,* isolated from human nares, produces a cyclic antimicrobial lugdunin. Both *S. lugdunensis* and lugdunin alone can reduce *S. aureus* nasal carriage (15). Additionally, common skin commensals *Staphylococcus epidermidis* (16), *Staphylococcus hominis* (17), and *Staphylococcus capitis* (18) have all been shown to have antagonistic activity against *S. aureus. Cutibacterium acnes* produces a thiopeptide antibiotic, cutimycin, which inhibits staphylococcal species to shape the hair follicle microbiome (19). Commensal staphylococcal species also produce bacteriocins, antimicrobial peptides that are believed to enable competition in the microbial community while protecting against MRSA and other pathogens (20). With the looming threat of antimicrobial resistance, harnessing mechanisms of interspecies competition within the skin microbiota could identify novel antimicrobials.

We previously showed that the pig skin microbiota contains a wide phylogenetic range of bacteria that inhibit MRSA *in vitro*. Pig skin is not only an established *in vivo* model for human skin (21), but pigs can also become stably colonized with *S. aureus* and MRSA, predominantly livestock-associated strains of MRSA (LA-MRSA) (10). Livestock colonization is a growing economic and public health concern; livestock workers, particularly swine workers (10), have high rates of nasal MRSA colonization by LA-MRSA, which can lead to subsequent LA-MRSA infections. The most effective method for reducing MRSA colonization in swine herds is the eradication of infected herds (22); however, this creates a large operational and economic burden.

The pig skin microbiome comprises a distinct microbial community from the human skin microbiome. At the genus level, the pig skin microbiome contains *Aerococcus*, *Rothia,* and *Kocuria* species that are not commonly found on human skin. At the species level, the pig skin microbiome contains a distinct set of coagulase-negative *Staphylococci* (CoNS), such as *Staphylococcus equorum* and *Staphylococcus haemolyticus*, which are non-overlapping with the CoNS found on human skin (23). We hypothesized that this distinct microbial community would uncover novel inhibitory mechanisms against *S. aureus*, which may be useful both for therapeutic purposes and for new understanding of interspecies competition in the skin microbiome.

Our previous work identified 37 unique bacterial species that inhibited MRSA *in vitro*, including 31 species that were not CoNS. We found that three of these pig isolates*—Aerococcus viridans, Rothia aerolata,* and *Desemzia incerta*—cooperatively exhibited colonization resistance against MRSA in a murine skin model (24). Here, we focused on one of these species, *D. incerta*, and investigated more deeply the molecular mechanisms of its interactions with *S. aureus*. We found that *D. incerta* supernatants exhibit MRSA-inhibitory activity *in vitro*. We further found that, at high inoculum, *D. incerta* precolonization exhibits MRSA colonization resistance *in vivo* in a murine skin model. Through a combination of genomic and proteomic approaches, we discovered that *D. incerta* exerts its antimicrobial activity through a secreted antimicrobial protein and identify a peptidoglycan hydrolase protein that is enriched in conditions of high antimicrobial activity. Using transcriptional profiling of *S. aureus* and *D. incerta* cocultures, we discovered that *S. aureus* exposure to *D. incerta* results in a significant decrease in *S. aureus* biofilm production. These studies provide greater insight into the microbial and molecular factors underlying resistance to *S. aureus* colonization on the skin and suggest that a peptidoglycan hydrolase might reduce *S. aureus* colonization.

## RESULTS

### A *Desemzia incerta* strain isolated from porcine skin inhibits MRSA via secreted product

**Fig 1:**
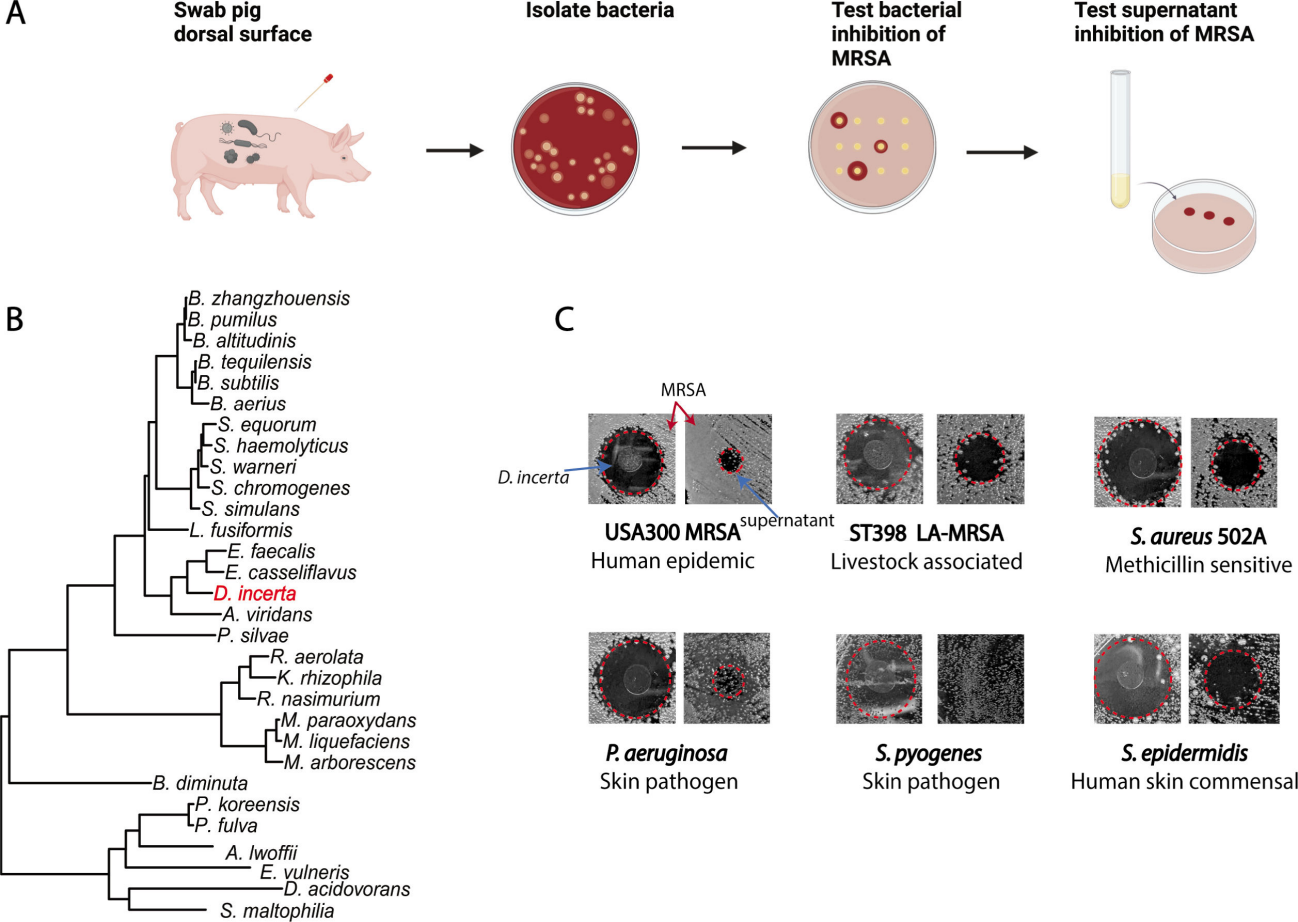
High-throughput screen of pig skin inhibitors reveals *Desemzia incerta* as an inhibitor of MRSA. (**A**) Strategy for screening pig skin isolates for inhibitors with secreted MRSA-inhibitory activity. (**B**) Dendrogram of unique MRSA-inhibitory bacterial identified from high-throughput screen. Phylogeny was constructed using representative 16S rRNA sequences curated from the Silva database (26). (**C**) Agar diffusion assay of *D. incerta* total cell culture (left) or concentrated supernatant (right) against various target skin pathogens and skin commensals. The edge of the zone of inhibition is demarcated in red dotted lines.

We previously cultured three bacterial isolates from porcine skin, *A. viridans*, *R. aerolata,* and *D. incerta,* that exhibited inhibitory activity against MRSA *in vitro* and together *in vivo* (24). To examine the individual mechanisms of inhibition, we tested whether these three isolates secreted antimicrobial molecules. We collected and concentrated cell-free supernatants from bacterial cultures and tested these supernatants for antimicrobial activity using an *in vitro* agar diffusion assay (Fig. 1A). Cell-free supernatants were concentrated via a 30-kDa molecular weight cutoff (MWCO) filtration. Live cell culture or concentrated supernatants were then spotted on a lawn of MRSA. The presence of a zone of clearing around the spot culture suggested MRSA growth inhibition. Of the three isolates tested, only the supernatant from *D. incerta*, a poorly studied Gram-positive rod (25) (Fig. 1B), inhibited MRSA (Fig. 1C). This suggests that *D. incerta* secretes an antimicrobial product. We also found that *D. incerta* cells and supernatant inhibited various staphylococcal strains, including human-epidemic MRSA (USA300), livestock-associated MRSA (ST398), and methicillin-sensitive *S. aureus* (strain 502A), as well as the common skin commensal *S. epidermidis*. We also observed inhibition against the Gram-negative skin pathogen *Pseudomonas aeruginosa* (Fig. 1C). While the skin pathogen *Streptococcus pyogenes* was inhibited by *D. incerta* live cell cultures, it was not inhibited by the *D. incerta* supernatant. Together, these findings suggest that *D. incerta* secretes an antimicrobial product with activity against a range of skin pathogens.

### *D. incerta* decreases MRSA colonization in a murine skin model

**Fig 2:**
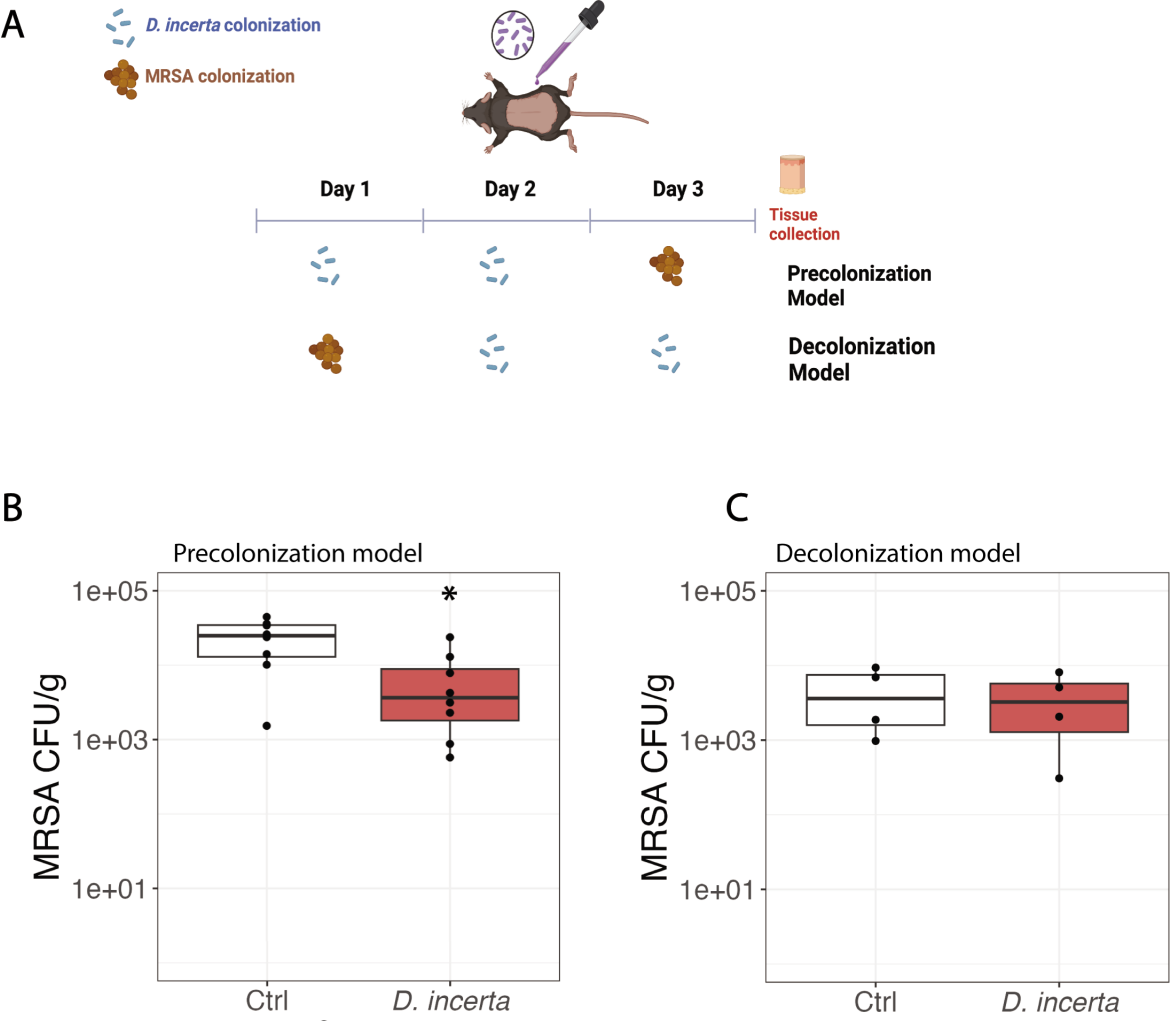
*D. incerta* exhibits colonization resistance against methicillin-resistant *Staphylococcus aureus*. (**A**) Experimental design of *in vivo* murine colonization experiments. Mice were shaved and colonized with *D. incerta* for 2 days or MRSA for 1 day in a precolonization or decolonization model. Three 6-mm punch biopsies were collected from the mouse dorsal surface, and the MRSA bacterial load was measured via MRSA-selective CHROMagar. (**B**) MRSA bacterial burden after *D. incerta* precolonization. (**C**) MRSA bacterial burden after *D. incerta* decolonization. *t*-test, ^*^P < 0.05.

We next investigated the ability of *D. incerta* to exert colonization resistance against MRSA in a murine skin colonization model on C57BL/6 mice (Fig. 2A). We examined both a precolonization (Fig. 2B) and decolonization (Fig. 2C) model, where *D. incerta* was either applied prior to (precolonization) or after (decolonization) MRSA challenge. In both models, 10^9^ CFU *D. incerta* was applied daily for 2 days and 10^8^ CFU USA300 MRSA was applied daily for 1 day. We found that precolonization with *D. incerta* reduced subsequent MRSA colonization by 60%, but decolonization with *D. incerta* did not have a noticeable effect on MRSA bacterial burden.

This result differs from our previously reported data, where precolonization by *D. incerta* was not sufficient to reduce MRSA colonization but showed a trend toward statistical significance (24). In our previous model, we used a lower inoculum of *D. incerta* (10^8^ CFU) on a different genetic background (SKH1 hairless mice). Altogether, the data suggest that mono-colonization with *D. incerta* exhibits colonization resistance against MRSA at high inocula, and using a consortium of bacteria as described previously (24) may increase this anti-MRSA effect.

### Parallel genomic and biochemical analyses of *D. incerta* supernatant reveal candidate antimicrobial proteins

To better understand the molecular factors underlying *D. incerta* inhibition of MRSA, we focused on the secreted antimicrobial factor found in *D. incerta* supernatant using a combination of genomic and biochemical approaches. First, we assembled a complete whole genome sequence of *D. incerta* using a hybrid sequencing approach combining Oxford Nanopore long reads and Illumina short reads. This approach allowed us to construct a *de novo* assembly of a complete, circular *D. incerta* genome which included five circular plasmids (27). We used antiSMASH v6 (28) to mine this genome for biosynthetic gene clusters (BGCs) encoding antimicrobial products. antiSMASH employs a homology-based search against a large database of known biosynthetic gene clusters (29). Fourteen BGCs were identified, all on the bacterial chromosome, and were predicted to encode nine saccharides, two fatty acids, two terpenes, and one type 3 polyketide (Table S1).

To support the genomic analysis, we performed biochemical characterization of the antimicrobial activity present in culture supernatants from *D. incerta*. The antimicrobial activity found in cell-free conditioned media failed to pass through a 50-kDa molecular weight cutoff filter and was eliminated after treatment with proteases trypsin or proteinase K (Fig. S1). These experiments suggested that the antimicrobial activity observed in *D. incerta* supernatant arose from an active protein of >50-kDa MW. As our genomic analysis did not predict any BGCs encoding peptide products, it is possible that *D. incerta* produces an antimicrobial protein not encoded by a known BGC.

**Fig 3:**
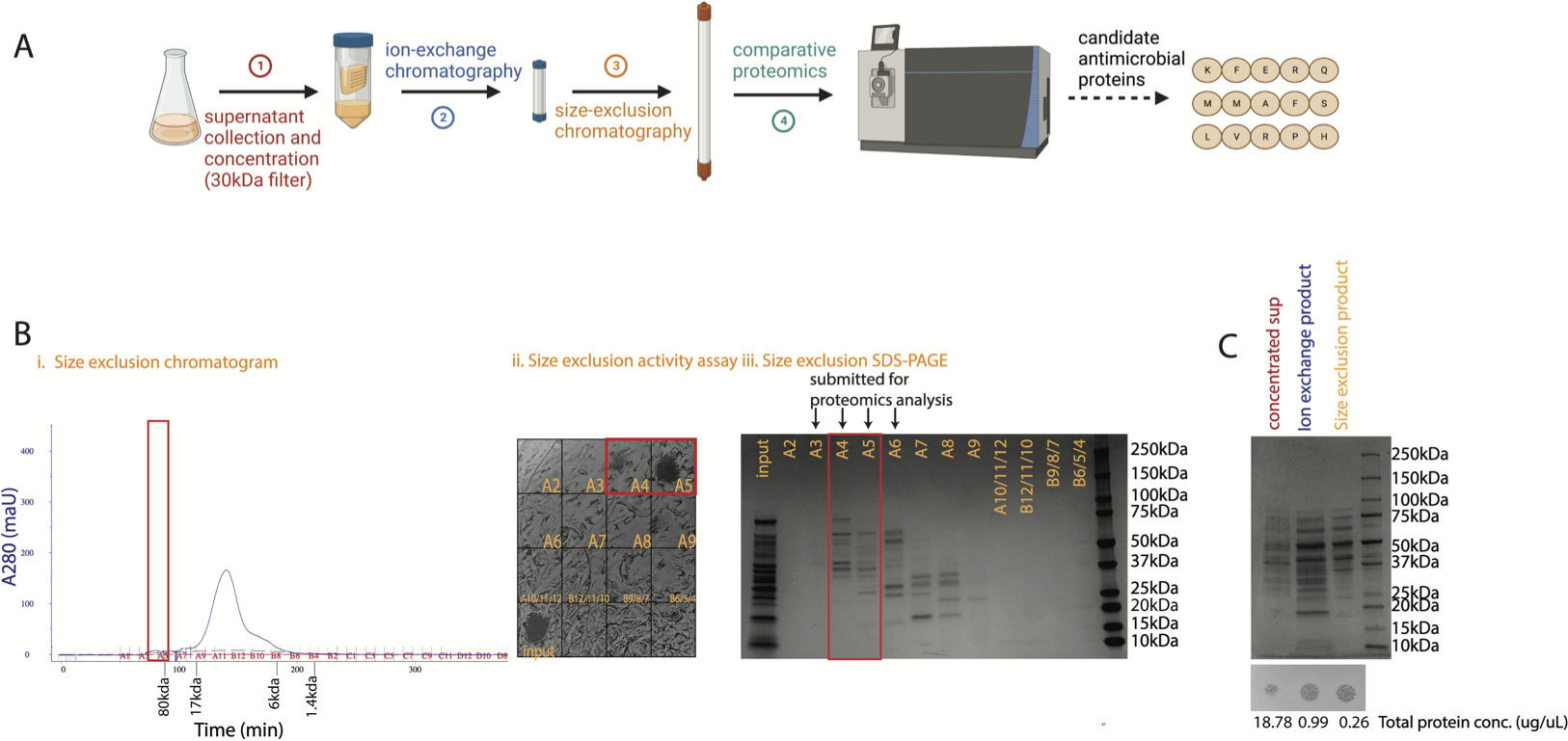
Protein purification and comparative proteomics yield candidate antimicrobial proteins. (**A**) Biochemical purification scheme used to generate candidate antimicrobial proteins. Three biological replicates were submitted for proteomics analysis. (**B**) Representative results from the final protein purification step (size exclusion chromatography). One biological replicate is shown. Active fractions, marked in red, are tracked via chromatogram (i), agar diffusion assay against *S. epidermidis* (ii), and SDS-PAGE (iii, 9 µL from each sample). Chromatography product from active fractions (**A4 and A5**) along with neighboring inactive fractions (**A3 and A6**) was submitted for proteomics analysis. (**C**) Representative results across all sequential protein purification steps (molecular weight fractionation, ion exchange chromatography, and size exclusion chromatography). One biological replicate is shown. SDS-PAGE (top, 30 µL from each sample), agar diffusion assay against USA300 MRSA (middle), and total protein concentration via NanoDrop A280 (bottom) are tracked for pooled active fractions from each purification step.

We next developed a protein purification strategy to isolate the antimicrobial activity from conditioned media (Fig. 3A). We combined molecular weight cutoff concentration with ion exchange and size exclusion chromatography to fractionate *D. incerta* cell-free supernatants. The agar diffusion assay described above was used to track activity throughout the purification scheme. After confirming that pooled chromatography products maintained activity against USA300 Rosenbach strain *S. aureus* (Fig. 3C), we switched to assaying individual chromatography fractions against *S. epidermidis* (Fig. 3B). *S. epidermidis* is a human skin commensal that is safer and more efficient to work with compared to MRSA. At each step, active fractions were pooled and used as the input for subsequent purification steps (Fig. 3A). This purification technique yielded two active fractions after the final chromatography step (Fig. 3B). The combined purification scheme resulted in an enrichment for antimicrobial activity; at each step, we noted a reduction in total protein concentration coincident with increased or stable antimicrobial activity as measured by the agar diffusion assay (Fig. 3C).

We submitted the resulting two active fractions from size exclusion chromatography, along with two flanking inactive fractions, for global proteomics analysis to identify proteins that were enriched in the fractions with antimicrobial activity. As *D. incerta* is a poorly studied bacterial species without an empirically validated reference proteome, we constructed a theoretical reference proteome based on predicted open reading frames within the *D. incerta* genome. This approach yielded 24 candidate proteins that were identified as enriched in the active antimicrobial fractions compared to inactive fractions (Table 1). Of particular interest was a putative peptidoglycan hydrolase protein; this class of enzyme can cleave peptidoglycan bonds in Gram-positive and Gram-negative (30) bacterial cell walls and has been demonstrated before to have antimicrobial activity. For example, lysozyme and lysostaphin, which have both been shown to have antistaphylococcal activity, are notable members of this family of enzymes (31). Lysostaphin has also been shown to disrupt *Staphylococcal* biofilms in addition to cleaving peptidoglycan bonds in the cell wall (32).

**TABLE 1:** Comparative proteomics reveals candidate antimicrobial proteins^*a*^

Protein names	Gene names	Length (aa)
Peptidoglycan hydrolase		1,018
Uncharacterized protein YaaQ		109
L-Lactate dehydrogenase		323
Alanine dehydrogenase		370
Aspartyl/glutamyl-tRNA(Asn/Gln) amidotransferase subunit B	gatB	476
6-Phospho-beta-glucosidase		476
D-Alanine–D-alanine ligase	ddl	369
Probable DNA-directed RNA polymerase subunit delta	rpoE	211
Ribonuclease J	rnj	556
tRNA uridine(34) hydroxylase	trhO	321
Serine hydroxymethyltransferase	glyA	412
Uracil phosphoribosyltransferase	upp	209
Mn-containing catalase		274
DEAD-box ATP-dependent RNA helicase	cshA	518
GMP reductase	guaC	324
NAD(P)H-dependent FMN reductase		184
Uridine kinase	udk	211
3-Oxoacyl-[acyl-carrier protein] reductase		261
23S rRNA (guanosine2251-2′-O)-methyltransferase		283
Formate–tetrahydrofolate ligase	fhs	556
DNA-binding transcriptional regulator, MarR family		153
Putative competence-damage inducible protein	cinA	419
Phosphate ABC transporter ATP-binding protein, PhoT family		254
Uncharacterized protein		346

^*a*^ Name, gene symbol, and length in amino acids of 24 proteins found to be enriched in active antimicrobial fractions after protein purification. Ribosomal subunits (e.g., 30S ribosomal subunit) were removed as low-confidence hits. Rows are unsorted.

### Transcriptional response of *S. aureus* to *D. incerta* exposure

**Fig 4:**
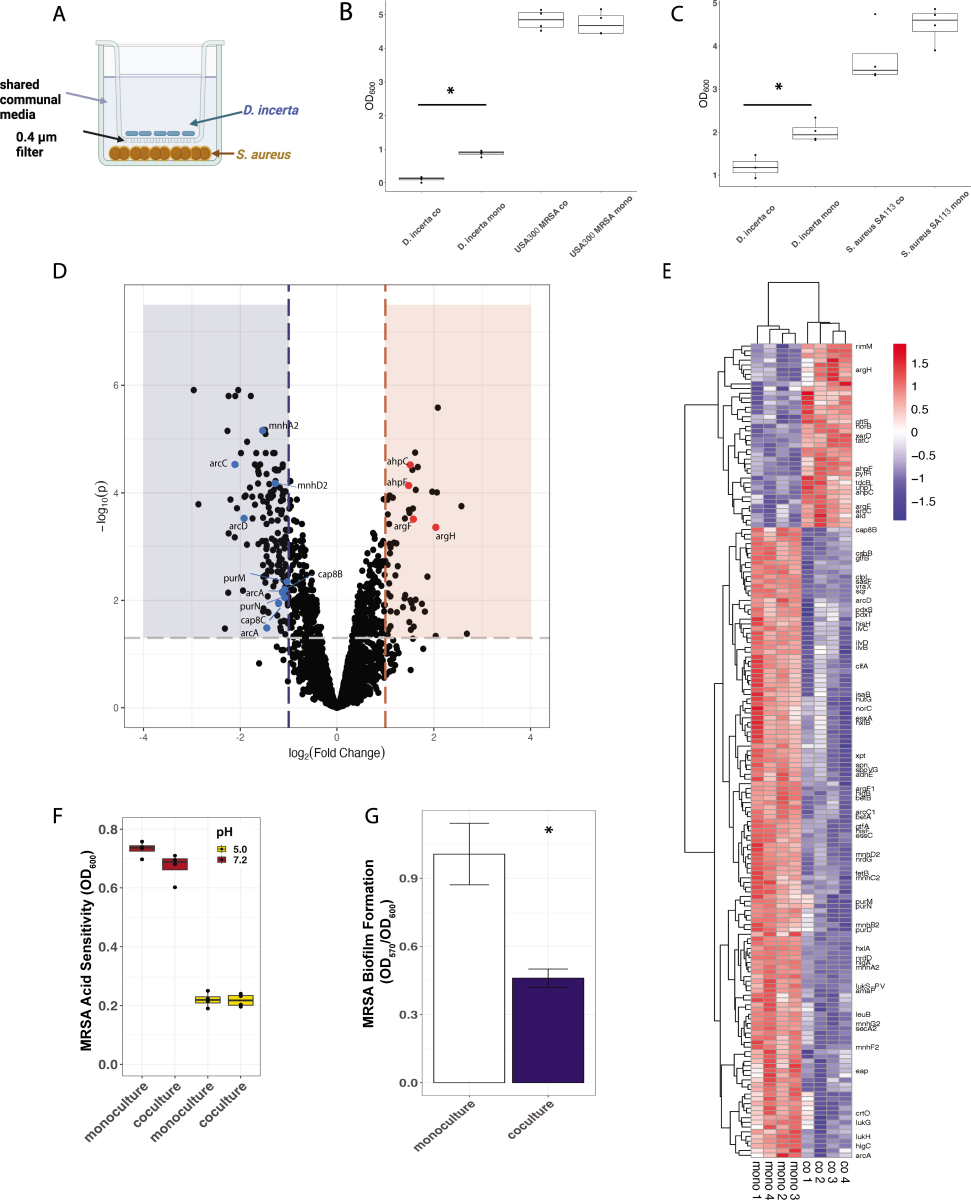
Transcriptional profiling of *S. aureus* reveals changes in biofilm formation after exposure to *D. incerta*. (**A**) Transwell coculture experimental design. Four wells per condition were extracted for RNA-seq analysis. (**B**) Bacterial density of *D. incerta* and *S. aureus* SA113 after 24 hours of incubation (OD_600_ of bacterial cultures before RNA extraction). (C) Bacterial density of *D. incerta* and USA300 MRSA after 24 hours of incubation (OD_600_ of bacteria before RNA extraction). (D) Volcano plot of genes in USA300 MRSA that were differentially expressed during *D. incerta* coculture compared to monoculture. Shaded areas highlight genes whose adjusted *P* < 0.05 and fold change >2.0 (blue, downregulated; red, upregulated). Bold points mark genes of interest, which were found within operons containing multiple differentially expressed genes. (**E**) Heatmap depicting differentially expressed genes in USA300 MRSA with adjusted *P* < 0.05 and fold change >2.0. For visualization purposes, genes were ordered by hierarchical clustering based on Pearson correlation. Values were scaled to the row mean. (**F**) USA300 MRSA cell density in response to acidified media (pH = 5.0, yellow) compared to neutral pH media (red) and coculture with *D. incerta*. Four wells per condition were used. (**G**) USA300 MRSA biofilm formation measured via crystal violet stain retention (OD_570_) normalized to cell growth (OD_600_). Two replicates of three wells per condition were used. *t*-test, ^*^P < 0.05.

In addition to investigating the mechanisms by which *D. incerta* exerts its antimicrobial activity, we also explored the response of *S. aureus* to *D. incerta*. We cocultured *D. incerta* with either SA113 strain *S. aureus* (methicillin-sensitive) or USA300 strain MRSA in a transwell coculture system (Fig. 4A). *D. incerta* liquid cultures were inoculated into the upper transwell and *S. aureus* cultures into the lower transwell at a ratio of 20 *D. incerta*:1 *S*. *aureus*. This ratio is comparable to the one used in our *in vitro* agar diffusion assay and our *in vivo* murine colonization studies. The bacterial cultures were separated by a 0.4-µm filter, which separates cells but allows for the transfer of diffusible molecules through a shared communal media. After 24 hours of growth, we measured cell density via optical density at 600 nm (OD_600_) and extracted RNA for transcriptomic analysis. We found that the *D. incerta* concentration used in this system resulted in a sublethal response by both *S. aureus* strains (Fig. 4B and C). Exposure to *D. incerta* led to a 17% reduction in culture density in *S. aureus* SA113 and no appreciable difference in culture density in USA300 MRSA. *D. incerta* cell density was significantly decreased by >40% in the coculture with both *S. aureus* strains, suggesting a codirectional competition between *D. incerta* and *S. aureus*.

We first examined the transcriptional interaction between *D. incerta* and USA300 MRSA, as this MRSA strain was the focus of our previous *in vitro* and *in vivo* experiments. We compared the transcriptional profile of USA300 strain *S. aureus* grown in coculture with *D. incerta* compared with *S. aureus* grown alone. We focused on the transcriptional response of USA300 MRSA, with particular interest in operons with multiple genes whose transcription was altered in the same direction. One hundred fifty-one genes were significantly downregulated with at least a twofold change in gene expression (Table S2), including multiple genes in the arc operon (*arcA*, *arcC*, and *arcD*), the pur operon (*purD*, *purH*, *purM*, and *purN*), the mnh family (*mnh*A-G), the luk family (*lukG, lukH,* and *lukS-PV*), and the cap family (*capA*, *cap8B*, and *cap8C*) (Fig. 4D and E). The arc operon encodes a constitutive arginine deaminase system that allows USA300 strain *S. aureus* to survive in acidic microenvironments such as the skin (33). The pur operon encodes purine biosynthesis (34); the synthesized purines can then be integrated into downstream nucleic acid synthesis. Mutations in *purM* have been associated with increased susceptibility to environmental stressors such as heat and acidity as well as increased susceptibility to the antibiotic rifampicin (35). The mnh proteins encode a family of Na^+^/H^+^ transporters that are essential for maintaining cytosol pH and enabling bacterial survival under high salt or high pH conditions (36).

The luk genes encode a family of *S. aureus* leukocidins, which are important *S. aureus* virulence factors that are well conserved across *S. aureus* lineages (37). Leukocidins are thought to protect *S. aureus* from phagocytosis by immune cells. Downregulation of these genes might, therefore, lead to decreased viability of *S. aureus in vivo*.

Capsular polysaccharide biosynthesis (cap) proteins are required for the synthesis of the polysaccharides that form the *S. aureus* capsule. Capsular polysaccharides have been found to protect *S. aureus* from phagocytic killing by human neutrophils (38). They are believed to be important in cellular adhesion and biofilm formation, though their role in these processes is still unclear: while capsular polysaccharide production is thought to inhibit bacterial adherence and biofilm formation in general, they may be important in maintaining mature biofilms (39). Defects in capsular polysaccharide synthesis have been associated with decreased nasal colonization of *S. aureus* (40).

Together, this transcriptional profile of downregulated genes in USA300 *S. aureus* suggests that exposure to *D. incerta* may result in greater sensitivity to environmental insults, such as acidic pH or high salt concentration, which might be present in the skin microenvironment. Downregulation of *cap* proteins might also lead to changes in biofilm production and decreased ability to colonize skin surfaces, while downregulation of leukocidins might decrease the evasion of host immune cells.

In USA300 MRSA*,* 55 genes were significantly upregulated after coculture with *D. incerta* (Table S3). This includes multiple genes in the ahp operon (*ahpC*, *ahpF*) and the arg operon (*argF*, *argH*) (Fig. 4D and E). ahpFC genes encode alkyl hydroperoxidase, which has been shown to play an important role in bacterial peroxide resistance (41). *argF* and *argH* are both genes involved in arginine biosynthesis (42). Notably, the concurrent downregulation of arginine catabolism genes (*arc* operon) and upregulation of arginine biosynthesis genes (*arg* operon) may represent a transcriptional response to low arginine. Taken together, the transcriptional profile of upregulated genes suggests that coculture with *D. incerta* may exert metabolic or oxidative stress onto *S. aureus*.

Though we observed different growth phenotypes between USA300 MRSA and *S. aureus* SA113 after exposure to *D. incerta* (18% growth decrease in SA113 and no growth decrease in USA300 MRSA), we found a remarkably similar transcriptional profile between *S. aureus* SA113 and USA300 MRSA after *D. incerta* exposure (Tables S4 and S5). Notably, we found a similar downregulation of genes in the arc operon, pur operon, and cap family of genes as well as a similar upregulation of the arg operon.

### Exposure to *D. incerta* decreases *S. aureus* biofilm formation

Because exposure to *D. incerta* led to downregulated *S. aureus* genes involved in pH response and capsule formation, we theorized that exposure to *D. incerta* leads to oxidative or metabolic stress in *S. aureus,* which may hamper the ability of *S. aureus* to survive in acidic conditions or maintain biofilms. We used the same transwell coculture system as above to test this hypothesis. First, we cocultured MRSA and *D. incerta* in acidified (pH = 5.0, approximately skin physiologic pH) media compared to standard (pH = 7.2) media. Though acidified media stunted MRSA growth compared to neutral pH media, we did not observe increased acid susceptibility after the coculture with *D. incerta* (Fig. 4F). Next, we measured MRSA biofilm formation under neutral pH by quantifying the retention of crystal violet stain (43). We noticed a dramatic decrease in MRSA biofilm formation after exposure to *D. incerta* (Fig. 4G).

We also noted a similar response to acidified media and a significant reduction in biofilm formation by *S. aureus* strain SA113 after exposure to *D. incerta* (Fig. S2).

Finally, we tested MRSA resistance to hydrogen peroxide after exposure to *D. incerta,* as we noticed an increase in expression of MRSA *ahpFC* alkylperoxidase genes after coculture with *D. incerta*. Five individual *S. aureus* cultures were cocultured with either *D. incerta* or tryptic soy broth (TSB) control using the coculture system described. After 24 hours of *D. incerta* exposure, the *S. aureus* cultures were challenged with hydrogen peroxide, and growth was tracked for an additional 24 hours. Four out of five replicates in the *D. incerta*-exposed group survived 0.8-mM hydrogen peroxide challenge, whereas only one out of five replicates in the control group survived hydrogen peroxide challenge (Fig. S3). This result suggests that exposure to *D. incerta* leads to an increased oxidative stress response in *S. aureus*.

## DISCUSSION

The phenomenon of colonization resistance is frequently described (44, 45) but still poorly understood. Better insight into the microbial interactions that shape this phenomenon is important on multiple fronts; first, it can provide greater clarity into the ways that microbial communities behave, which can have important implications for human health (46, 47). Secondly, competitive microbial interactions can be tapped for their therapeutic potential, enabling the discovery of novel antibiotic compounds for which there is an urgent need (48). Here, we focus on a single competitive interaction between the pig skin isolate *D. incerta* and human-epidemic strain USA300 MRSA, which we show to exhibit colonization resistance on murine skin. We examined the factors that *D. incerta* uses to initiate this interaction, as well as the response by methicillin-resistant *S. aureus*. We identify 24 candidate antimicrobial proteins secreted by *D. incerta* that might be responsible for its anti-MRSA activity. Of particular interest is a secreted peptidoglycan hydrolase protein, which has been previously proposed to have antimicrobial function. We further profiled the transcriptional response by *S. aureus* and found a diminished expression of capsular synthesis genes concurrent with a decrease in biofilm production. These factors might contribute to the colonization resistance phenotype we observe *in vivo*.

Notably, *D. incerta* exhibited a different antimicrobial pattern against *S. pyogenes* than against *S. aureus* (Fig. 1C), which points to the presence of additional antibiotic factors separate from the antimicrobial proteins we focused on here. For example, our genomic analysis indicates that the *D. incerta* genome encodes a type 3 polyketide synthesis (T3PKS) biosynthetic gene cluster (Table S1). Polyketides comprise a diverse range of natural products, many of which are antimicrobial (49). They would also not be expected to pass through the molecular weight cutoff filter we used in our experiments.

While *S. aureus* exposure to *D. incerta* cells and supernatant resulted in clear growth reduction on a solid agar diffusion assay, as demonstrated by a zone of clearing, this growth defect was not recapitulated when *S. aureus* was cocultured with *D. incerta* in liquid culture in our transwell experiments. This difference may be the result of the different nutrient conditions, spatial organization, or culture timing present between the two *in vitro* models we used. The transcriptional profile of *S. aureus* in liquid coculture pointed to a placation in *S. aureus* virulence through reduced immune evasion (*luk* genes) and decreased capsule synthesis (*cap* genes). Notably, these changes were present in both USA300 MRSA and SA113 *S. aureus* despite USA300 MRSA not exhibiting a decrease in cell density, suggesting that this response is not correlated with cell growth. It is, therefore, unclear whether the reduction in *S. aureus* colonization we observed *in vivo* occurred primarily through a reduction in *S. aureus* burden through bacterial antimicrobial factors or through decreased *S. aureus* immune evasion and colonization efficiency.

We observed a decrease in *D. incerta* growth during liquid coculture with both USA300 MRSA and SA113 *S. aureus*, suggesting that *S. aureus* can also inhibit *D. incerta* under certain conditions. Our transcriptional profile of *S. aureus* highlighted multiple changes in *S. aureus* nutrient metabolism, particularly arginine metabolism, suggesting that the growth decrease in *D. incerta* may be due to competition for limiting nutrients such as arginine. *S. aureus* is also known for producing a number of bactericidal proteins (50); though we did not notice a change in transcription of these bacteriocins during *D. incerta/S. aureus* coculture, release of toxic metabolites from constitutive *S. aureus* expression or lysis of *S. aureus* cells may also contribute to *D. incerta* killing.

We focused our studies on a single strain of *S. aureus*, USA300 methicillin-resistant *S. aureus*, because of its high healthcare burden as the leading cause of human-epidemic MRSA infections in the United States (51). Nonetheless, we also observed antimicrobial activity by *D. incerta* against multiple other *S. aureus* strains, including livestock-associated ST398 strain MRSA. Thus, microbiome-derived antimicrobial products might be useful not only for human health but also for reducing the MRSA burden among livestock, which can have both agricultural and public health benefits.

Pigs are an economically important livestock animal that exhibits high rates of MRSA colonization. Pigs and other livestock animals can act as a reservoir for antimicrobial resistance that can cause infection in humans, thus exacerbating an existing public health concern (52). Here, we paradoxically exploited the propensity for MRSA colonization in pigs for therapeutic benefit, by exploring the diverse microbial community on pig skin to identify novel MRSA inhibitors. *D. incerta*, apart from being poorly studied in general, is unique in that it is one of the few MRSA inhibitors isolated from the skin microbiome that is not also a *Staphylococcus* species. A candidate antimicrobial molecule secreted by *D. incerta* that we identified is also much larger (80 kDa based on size exclusion chromatography) than other antimicrobial molecules isolated from human skin (15, 16). The uncovering of *D. incerta*, of which relatively little is known, and its secreted antimicrobial products demonstrates the benefit of exploring alternative microbial communities for new products.

The human microbiome undergoes dynamic exchange with environmental exposures, including other microbial communities. In the context of the skin microbiome, this means that humans may experience the transfer of pathogenic bacteria such as *S. aureus* through domestic or occupational exposure, including livestock exposure. This transfer is particularly pronounced between swine, likely due to the similarities between porcine and human skin. Here, we show that this shared burden of *S. aureus* colonization can also provide an opportunity for therapeutic discovery and understanding of microbial competition.

## MATERIALS AND METHODS

### *D. incerta* culture and supernatant collection

*D. incerta* from glycerol stocks was streaked onto a blood agar plate and incubated overnight at 37°C. A single large colony was inoculated into 3-mL TSB and incubated overnight at 37°C without shaking. This 3-mL culture was subinoculated into 50-mL TSB and incubated overnight at 37°C without shaking. The 50-mL culture was subinoculated into 500–1,000-mL TSB and incubated at room temperature for 3 days without shaking. Cells were pelleted via centrifugation at 15,000 *× g* for 15 min, and the supernatant was filtered through a Stericup 0.22-µm sterile filter. Cell-free supernatants were concentrated to <50-mL total volume using an Amicon stirred cell model 8050 fitted with a 76-mm 30-kDa Ultracel MWCO filter (Amicon) or a 76-mm 50-kDa Biomax MWCO filter (Amicon).

### Agar diffusion assay

*Staphylococcus aureus* was inoculated into 3-mL TSB and incubated at 37°C overnight without shaking. Overnight cultures were diluted to a final OD_600_ of 0.1. One hundred microliters of dilute culture was spread onto a 7-cm tryptic soy agar plate using glass beads. Five microliters of undiluted *D. incerta* culture, concentrated *D. incerta* supernatant, or chromatography product was spotted on the *S. aureus* lawn. The plate was then incubated overnight at 37°C.

### Construction of phylogenetic tree

Representative 16S ribosomal RNA sequences for each species were curated from the Silva Living Tree Project database. For species without a Living Tree Project entry, the longest high-quality sequence from the Reference Non-Redundant data set was used.

Multiple sequence alignment of representative 16S rRNA sequences was generated using MAFFT (53) v. 7.505 using the L-INS-i setting. A maximum likelihood tree was constructed from the multiple sequence alignment with RAxML (54) v. 8.2.12 using the best tree from 100 searches. The tree was midpoint rooted in FigTree (55) v. 1.4.4 and visualized in RStudio.

### Whole genome sequencing and assembly

Genomic DNA was extracted from *D. incerta* cultures using the ZymoResearch QuickDNA Fungal/Bacterial MicroPrep kit. Illumina library preparation and sequencing were performed by the CHOP Microbiome Center, using the Illumina DNA Prep kit and unique dual indexes (Illumina Nextera Index kit) at a 1:4 scale reaction volume, and sequenced on the IlluminaHiSeq2500. Trim-galore (56) v. 0.2.4 was used to trim adapter sequences. Quality control of reads was performed before and after read trimming using fastQC (57) v. 0.11.8 settings. In tandem, high-molecular-weight genomic DNA was extracted using the NEB Monarch HMW DNA Extraction Kit for Tissue using the standard input workflow for Gram-positive bacteria. For lysis, 80 µL of 100 mg/mL lysozyme in STET buffer was used, with no other modifications to the manufacturer protocol. Library preparation and ONT sequencing of high-molecular-weight DNA were performed by SeqCenter. Libraries were prepared using the ONT Genomic DNA by Ligation kit and sequenced on an ONT MinION with R9 flow cells (R9.4.1). Base calling was performed using Guppy (58) v. 5.0.16 in a high-accuracy base calling mode. PoreChop (59) v. 0.2.4 was used to trim adapter sequences. Hybrid assembly using both long and short reads was constructed using Unicycler (58) v. 0.4.8. Genome annotation was performed by prokka v. 1.14.6 (60).

### antiSMASH analysis

The assembled genome fasta was used as input for antiSMASH (61) v7 in the interactive web browser at https://antismash.secondarymetabolites.org/ under “loose” settings with all extra features (KnownClusterBlast, ClusterBlast, SubClusterBlast, MiBiG cluster comparison, ActiveSiteFinder, RREFinder, Cluster Pfam analysis, Pfam-based GO annotation, TIGRfam analysis, and TFBS analysis) enabled.

### Ion exchange chromatography

A concentrated cell-free supernatant was prepared as described, and activity was validated against *S. epidermidis* by the agar diffusion assay. Bis-Tris buffer pH = 5.9 was added to the supernatant to 20 mM and loaded using 50 mL Superloop onto a 5-mL HiTrap QFF column (Cytiva) with gradient elution to 0.5 M NaCl in Bis-Tris buffer. Eight-milliliter fractions were collected and concentrated using a 30-kDA MWCO filter (Amicon). The activity of individual fractions was tested using agar disk diffusion against *S. epidermidis*. The activity of pooled active fractions was validated against USA300 MRSA.

### Size exclusion chromatography

Active fractions from the ion exchange column were pooled, concentrated, and applied to a HiPrep 16/60 S200 column (20 mM Bis-Tris pH = 5.9, 50 mM NaCl), and 4 mL fractions were collected. The fractions were concentrated using a 30-kDa MWCO filter (Amicon), and the activity of individual fractions was tested using agar disk diffusion against *S. epidermidis*. The activity of pooled active fractions was validated against USA300 MRSA.

### Preparation of fractions for mass spectrometry

Fractions from size exclusion chromatography were precipitated by adding NaCl to a final concentration of 100 mM, adding four volumes of room temperature acetone, and vortexing and incubating at room temperature for 30 min. Precipitated proteins were pelleted by spinning at 20k × *g* for 10 min (62). Pellets were solubilized and digested with the iST kit (PreOmics GmbH, Martinsried, Germany) as per the manufacturer’s protocol (63). Briefly, the resulting pellet was solubilized, reduced, and alkylated by the addition of sodium deoxychoate (SDC) buffer containing tris(2-carboxyethyl)phosphine (TCEP) and 2-chloroacetamide then heated to 95°C for 10 min. Proteins were enzymatically hydrolyzed for 1.5 hours at 37°C by the addition of LysC and trypsin. The resulting peptides were desalted, dried by vacuum centrifugation, and reconstituted in 0.1% trifluoroacetic acid (TFA) containing iRT peptides (Biognosys, Schlieren, Switzerland).

### Mass spectrometry data acquisition

Samples were analyzed on a Q-Exactive HF mass spectrometer (ThermoFisher Scientific, San Jose, CA) coupled with an UltiMate 3000 nano UPLC system and an EasySpray source. Peptides were loaded onto an Acclaim PepMap 100 75 µm × 2 cm trap column (Thermo) at 5 µL/min and separated by reverse phased (RP)-HPLC on a nanocapillary column, 75 µm id × 50 cm 2 µm PepMap RSLC C18 column (Thermo). Mobile phase A consisted of 0.1% formic acid and mobile phase B of 0.1% formic acid/acetonitrile. Peptides were eluted into the mass spectrometer at 300 nL/min with each RP-LC run comprising a 90-min gradient from 3% B to 45% B.

The mass spectrometer was set to repetitively scan m/z from 300 to 1,400 (*R* = 240,000) followed by data-dependent mass spectrometry/mass spectrometry (MS/MS) scans on the 20 most abundant ions, minimum automatic gain control (AGC) 1*e*4, dynamic exclusion with a repeat count of 1, repeat duration of 30 s, and resolution of 15,000. The AGC target value was 3*e*6 and 1*e*5, for full and MSn scans, respectively. The MSn injection time was 160 ms. The rejection of unassigned and 1+, 6–8 charge states was set.

### System suitability and quality control

The suitability of the Q Exactive HF instrument was monitored using QuiC software (Biognosys, Schlieren, Switzerland) for the analysis of the spiked-in iRT peptides. Meanwhile, as a measure of quality control, we injected standard *Escherichia coli* protein digest prior to and after injecting the sample set and collected the data in the data-dependent acquisition (DDA) mode. The collected data were analyzed in MaxQuant (64), and the output was subsequently visualized using the PTXQC (65) package to track the quality of the instrumentation.

### MS data processing and analysis

The raw files for DDA analysis were processed with FragPipe (66) version 20.0 using its default workflow. The reference *Desemzia incerta* proteome from UniProt (2,143 canonical and isoform proteins) was concatenated with the self-generated reference proteome and common protein contaminants and used for the search. The default MS1 quantification was performed using the IonQuant algorithm through FragPipe without the match between runs option. Data processing and statistical analysis were performed in R. The raw MS1 intensity data were log2-transformed and normalized by subtracting the median for each sample. After data filtering, the limma *t*-test was employed to identify differentially abundant proteins between high and no-activity fractions using *P*-value <0.05 as a significant threshold. The proteins that were exclusively detected in one experimental group were also reported for further bioinformatics analysis.

### Transwell cocultures

*S. aureus* and *D. incerta* overnight cultures were grown as described. *S. aureus* was diluted to a final OD_600_ of 0.05. *D. incerta* cultures were concentrated to a final OD_600_ of 2.0. One milliliter of dilute *S. aureus* culture or TSB control was loaded into the bottom well of a Corning transwell 12-well cell culture plate (0.4-µm pore size). Five hundred microliters of *D. incerta* culture or TSB control was loaded into the top well of the corresponding transwell insert. Plates were incubated at 37°C overnight without shaking. The contents of the bottom and top well were collected separately into 1.5-mL microcentrifuge tubes. One hundred microliters was reserved for OD_600_ measurements (model name, Eppendorf). The remainder was centrifuged at >10,000 × *g* for 1 min. The supernatant was decanted, and the cell pellet was stored at −80°C.

### Growth in acidified media

Tryptic soy broth was prepared according to manufacturer specifications and found to have pH = 7.2. No modifications were made to this standard TSB in the neutral pH condition. To create acidic media, concentrated acetic acid was added dropwise to standard TSB until the pH reached 5.0. *D. incerta* and *S. aureus* were seeded into transwell culture plates at the ratios described above in either standard media or acidic media and incubated overnight at 37°C. The top well containing *D. incerta* was removed, and OD_600_ of the *S. aureus* culture was measured on a BioTek Synergy HT microplate reader.

### Biofilm formation assay

*D. incerta* and *S. aureus* were seeded into transwell tissue culture plates as described above into TSB media containing 0.5% dextrose. Cultures were incubated for 24 hours at 37°C. The top well containing *D. incerta* was discarded, and *S. aureus* cultures in the bottom well were gently resuspended. The OD_600_ of the *S. aureus* was measured on a microplate reader, and the *S. aureus* cultures were discarded. The plate was rinsed vigorously in deionized water, and the wells were stained with 500 µL crystal violet solution. After 10 min, the crystal violet stain was removed, and the plate was rinsed vigorously in deionized water and allowed to dry for 5 min. The remaining crystal violet stain was solubilized in 500 µL 30% acetic acid, which was diluted in equal volume phosphate-buffered saline (PBS). The OD_570_ of the solubilized crystal violet was measured on a microplate reader.

### Hydrogen peroxide survival assay

*D. incerta* and *S. aureus* were seeded into transwell culture plates as described. Cultures were incubated for 24 hours at 37°C. The top well (containing *D. incerta*) was discarded, and *S. aureus* cultures in the bottom well were gently resuspended. *S. aureus* cultures were diluted to an OD_600_ of 0.01, and 90 µL of each replicate was transferred into a 96-well plate. The growth curves of the *S. aureus* replicates were recorded on a microplate reader prewarmed to 37°C and set to read OD_600_ every 12 min. Cultures were allowed to grow undisturbed for 90 min. Then, 10 µL of hydrogen peroxide was added to each well to achieve a final concentration of 0.8 mM hydrogen peroxide. We found this concentration to be close to the minimum inhibitor concentration for USA300 MRSA. Growth in hydrogen peroxide was recorded for a total of 24 hours.

### RNA extraction and sequencing

RNA from frozen bacterial cell pellets was extracted using the Zymo Research Direct-zol RNA purification kit. RNA sequencing was performed by SeqCenter using their standard RNA sequencing methods. In brief, samples were DNAse-treated with Invitrogen DNAse (RNAse-free). Library preparation was performed using Illumina’s Stranded Total RNA Prep Ligation with Ribo-Zero Plus kit and 10 bp IDT for Illumina indices. Sequencing was done on a NextSeq2000 giving 2 × 51 bp reads. Demultiplexing, quality control, and adapter trimming were performed with bcl-convert (v. 3.9.3).

### RNA sequencing analysis

Quality control of RNA reads was performed using fastqc (57) v. 0.11.9. Reads were mapped to coding sequences from the *S. aureus* representative genome NC_010079.1 using Kallisto (67) v. 0.48.0. Mapped genes were filtered and normalized using edgeR (68) v. 3.40.2 and limma (69) v. 3.54.2 packages in Rstudio v. 4.2.2. Gene ontology (GO) terms were assigned using the UniProt (70) IDmapping tool, and GO enrichment was performed using the goseq (71) v. 1.50.0 package in Rstudio (72).

### Mouse colonization resistance experiments

All mouse procedures were performed under protocols approved by the University of Pennsylvania IACUC. Seven-week-old female C57BL/6 mice were allowed to acclimate for 1 week before experimentation began. Mice were given *ad libitum* access to food and water. *D. incerta* was grown in 3-mL liquid TSB overnight at 37°C without shaking, then subinoculated into 50-mL TSB overnight at 37°C without shaking. MRSA isolates were grown overnight in TSB media at 200 rpm. On the following day, OD_600_ measurement was used to standardize inoculums, and pellets were resuspended in TSB to acquire 1 × 10^9^ CFU/mL inocula (1 × 10^8^ for MRSA). Mice were shaved across their entire back, and the cage was changed 1 day prior to initial colonization. No additional cage changes were performed throughout the duration of the experiment. The inocula of MRSA (10^8^) or *D. incerta* (10^9^) were prepared from overnight cultures resuspended in 100-µL TSB media using the conversion 1OD = 3.3*e*8 CFU/mL for *S. aureus* and 1OD = 3.5*e*8 CFU/mL for *D. incerta*. Mice were anesthetized, and inocula were pipetted onto their shaved backs and spread with a foam-tipped applicator. This procedure was repeated for 3 days with experimental groups as described. Twenty-four hours after the final inoculum was applied, mice were euthanized. Three-by-six-millimeter skin punch biopsies were collected into a preweighted Eppendorf tube containing 500 µL TSB and a 1/4″ ceramic bead (MP Biomedicals). Samples were weighed and agitated for 30 min on a Vortex-Genie 2 with horizontal tube attachment. One hundred microliters of liquid was spread onto a blood agar or MRSA-selective CHROMagar (*S. aureus* CHROMagar + 5 mg/mL cefoxitin) and incubated overnight at 37°C.

### Microbial strains

Strains will be made available upon request.

**Table:** 

Strain	Source
*Staphylococcus aureus* subsp. aureus Rosenbach. Strain TCH1516 [USA300-HOU-MR]	ATCC cat.# BAA-1717
*Staphylococcus aureus* 502A subsp. aureus Rosenbach	ATCC # 27217 serologic type (b)c1
*Staphylococcus aureus* S0385 (LA-MRSA)	BEI Resources (ATCC) Cat# NR-28983
*Streptococcus pyogenes* (strain designation: SF370; M1 GAS)	ATCC Lot # 70012149
*Staphylococcus epidermidis* (EGM 2-01)	Isolated from healthy human skin Initially described in Uberoi et al. (73)
*Pseudomonas aeruginosa* (DOERN #1896)	Isolated from diabetic foot ulcer Initially described in Gardner et al. (74)
*Aerococcus viridans* (EGM 1-16)	Isolated from porcine skin Initially described in Wei et al. (24)
*Rothia aerolata* (EGM 1-37)	Isolated from porcine skin Initially described in Wei et al. (24)
*Desemzia incerta* (EGM 1-39)	Isolated from porcine skin Initially described in Wei et al. (24)

## Data Availability

Genome sequences are available in NCBI databases under BioProject PRJNA974032, Biosample SAMN35158552, GenBank CP CP126128.1 (chromosome), NZ_CP126129.1 (plasmid 1), NZ_CP126130.1 (plasmid 2), NZ_CP126131.1 (plasmid 3), NZ_CP126132.1 (plasmid 4), NZ_CP126133.1 (plasmid 5), SRA SRR24674626 (long read), and SRR24710524 (short read). RNAseq data are available in the Gene Expression Omnibus (GEO) under series GSE239513.

## SUPPLEMENTAL MATERIAL

Supplemental material msphere.00636-23-s0001.docx Supplemental tables and figures.

ASM does not own the copyrights to Supplemental Material that may be linked to, or accessed through, an article. The authors have granted ASM a non-exclusive, world-wide license to publish the Supplemental Material files. Please contact the corresponding author directly for reuse.
