# Supplementary material for: An exploration of mechanisms underlying Desemzia incerta colonization resistance to methicillin-resistant Staphylococcus aureus on the skin
Source: mSphere. 2024 Feb 28;9(3):e00636-23. doi: 10.1128/msphere.00636-23 (PMC10964421; doi:10.1128/msphere.00636-23)
Supplement: Supplemental material — Supplemental tables and figures. [file msphere.00636-23-s0001.docx]

**SUPPLEMENTAL MATERIALS**


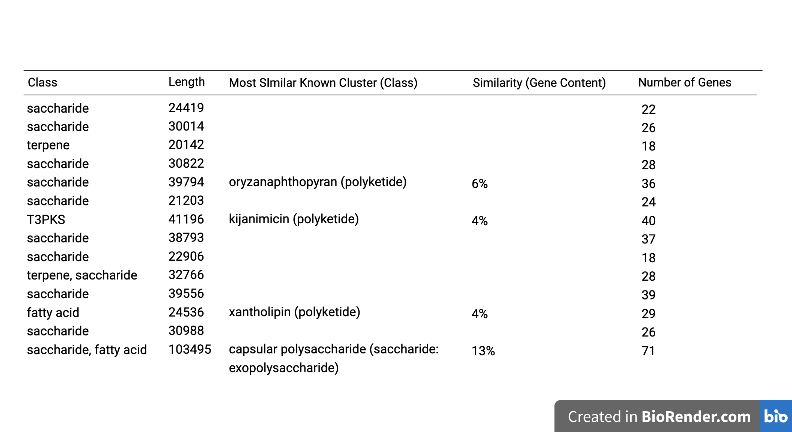
**Table S1: Summary of antiSMASH analysis of *D. incerta* complete genome**. ‘Loose’ setting was used with all additional features enabled.

**Figure S1: *D. incerta* supernatant is >50kDa and sensitive to protease digestion**. **A)** 5 μL *D. incerta* cell culture (OD ~ 1.0) was spotted onto a lawn of USA300 MRSA (100 μL, OD = 0.1) on Trypic Soy Agar (TSA) and incubated overnight at 37°C. **B)** Conditioned media was collected from *D. incerta* overnight cell cultures and concentrated 20x using a 50kDa molecular weight cutoff (MWCO) filter. The retentate was kept as >50kDa fraction, while the flow through was kept as <50kDa fraction. The flow through was then concentrated 20x in a 30kDa MWCO filter. The retentate was kept as >30kDa but <50kDa fraction, and the flow through was kept as
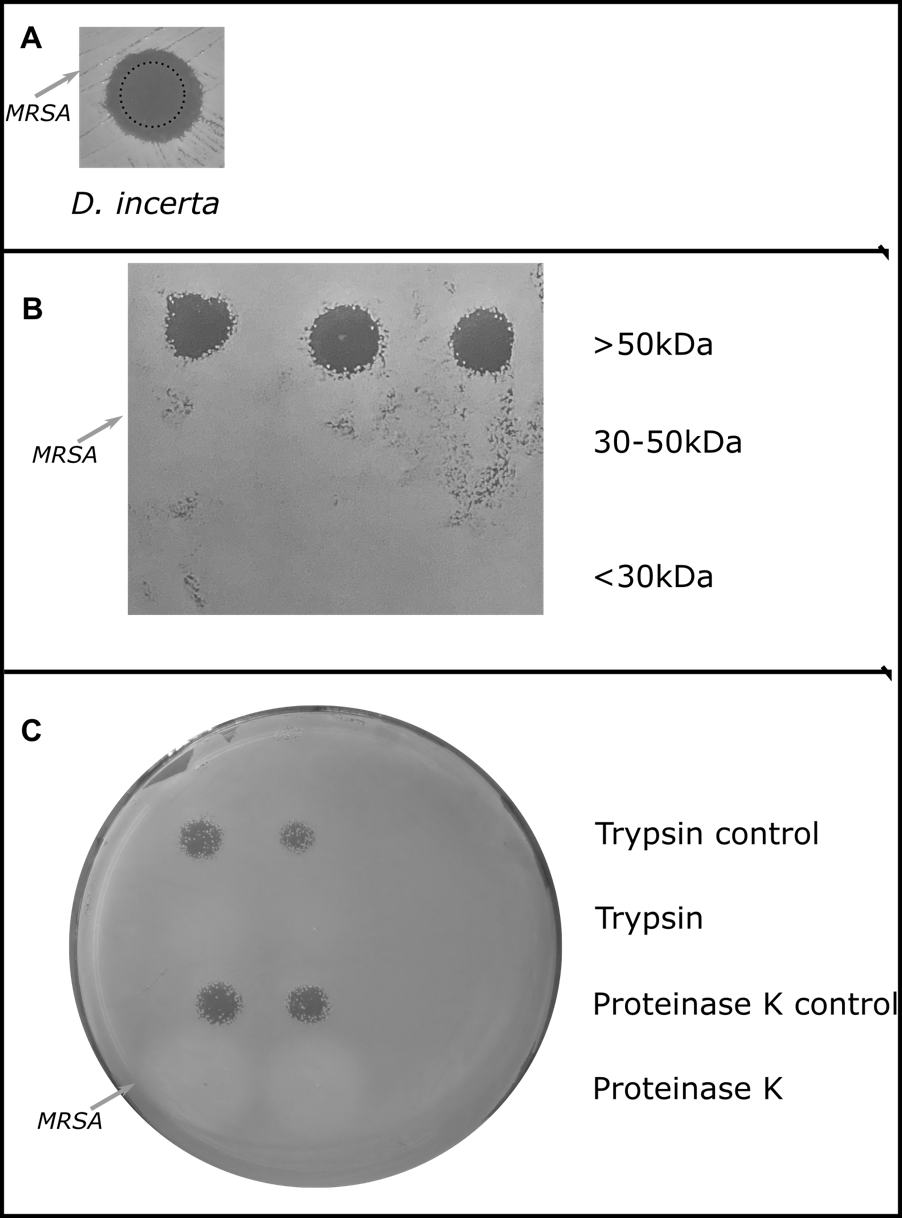
<30kDa fraction. Fractions were spotted on a lawn of MRSA as in (A). Rows represent technical triplicates. **C)** Concentrated supernatant was collected as described in (B). Concentrated supernatant with confirmed antimicrobial activity was incubated with either 60 BAEE units Trypsin, 20 mAU Proteinase K, or the corresponding buffer control for 4 hrs at 37°C. Protease treated samples were tested for MRSA inhibition via the agar diffusion assay described in A. Rows represent technical duplicates.

| **RefSeqID** | **log_2_(FC)** | **adj. p val** | **Gene Symbol** | **Protein** |
| --- | --- | --- | --- | --- |
| WP_000594519.1 | -2.96 | 1.2E-06 | hlgA | bi-component gamma-hemolysin HlgAB subunit A |
| WP_000669519.1 | -2.87 | 1.6E-04 | spn | myeloperoxidase inhibitor SPIN |
| WP_000595263.1 | -2.32 | 3.3E-02 |  | transcriptional activator RinB |
| WP_000601724.1 | -2.26 | 7.1E-06 | nrdG | anaerobic ribonucleoside-triphosphate reductase activating protein |
| WP_001549197.1 | -2.25 | 7.2E-03 |  | delta-lysin family phenol-soluble modulin |
| WP_000793596.1 | -2.24 | 1.6E-06 | argF | ornithine carbamoyltransferase |
| WP_025175201.1 | -2.24 | 5.7E-04 |  | hypothetical protein |
| WP_000730043.1 | -2.20 | 1.3E-04 |  | SDR family oxidoreductase |
| WP_000036757.1 | -2.12 | 3.0E-05 | arcC | carbamate kinase |
| WP_000475805.1 | -2.11 | 6.8E-04 |  | universal stress protein |
| WP_001071717.1 | -2.10 | 1.6E-06 | nrdD | anaerobic ribonucleoside-triphosphate reductase |
| WP_001093553.1 | -2.10 | 3.0E-05 |  | DM13 domain-containing protein |
| WP_001005511.1 | -2.07 | 1.9E-04 |  | fructosamine kinase family protein |
| WP_000777188.1 | -2.04 | 1.2E-06 |  | metal-dependent hydrolase |
| WP_001549607.1 | -1.99 | 1.8E-05 |  | MAP domain-containing protein |
| WP_000272781.1 | -1.94 | 6.6E-03 |  | Crp/Fnr family transcriptional regulator |
| WP_000239610.1 | -1.93 | 3.0E-04 |  | phospholipase |
| WP_001237629.1 | -1.91 | 1.2E-04 |  | hypothetical protein |
| WP_000791407.1 | -1.89 | 1.1E-04 | lukH | bi-component leukocidin LukGH subunit H |
| WP_000595324.1 | -1.86 | 9.2E-04 | lukG | bi-component leukocidin LukGH subunit G |
| WP_000752917.1 | -1.86 | 1.1E-05 |  | CsbD family protein |
| WP_001056223.1 | -1.80 | 8.0E-05 | clfA | MSCRAMM family adhesin clumping factor ClfA |
| WP_000206639.1 | -1.78 | 1.6E-06 |  | ArgE/DapE family deacylase |
| WP_000916713.1 | -1.75 | 1.2E-04 | hlgC | bi-component gamma-hemolysin HlgCB subunit C |
| WP_000344142.1 | -1.75 | 3.0E-04 | arcD | arginine-ornithine antiporter |
| WP_001789452.1 | -1.70 | 3.0E-05 |  | acyl CoA:acetate/3-ketoacid CoA transferase |
| WP_000558975.1 | -1.70 | 1.7E-04 |  | alpha/beta hydrolase |
| WP_001177186.1 | -1.67 | 1.8E-05 |  | hypothetical protein |
| WP_000066521.1 | -1.64 | 3.3E-05 | betA | choline dehydrogenase |
| WP_000036327.1 | -1.63 | 6.7E-05 |  | DUF1648 domain-containing protein |
| USA300HOU_RS16045 | -1.63 | 4.0E-05 | hisF | imidazole glycerol phosphate synthase subunit HisF |
| WP_000857159.1 | -1.62 | 9.0E-04 |  | zinc-dependent alcohol dehydrogenase family protein |
| WP_000783428.1 | -1.62 | 4.5E-05 | hlgB | bi-component gamma-hemolysin HlgAB/HlgCB subunit B |
| WP_000071901.1 | -1.59 | 3.0E-05 |  | pyridoxamine 5'-phosphate oxidase family protein |
| WP_001077722.1 | -1.58 | 1.2E-04 |  | YjiH family protein |
| WP_000708242.1 | -1.55 | 7.9E-04 |  | antibiotic biosynthesis monooxygenase |
| WP_001096791.1 | -1.53 | 2.2E-03 |  | lantibiotic protection ABC transporter ATP-binding subunit |
| WP_001008401.1 | -1.53 | 2.9E-04 |  | class I adenylate-forming enzyme family protein |
| WP_000446299.1 | -1.53 | 1.6E-02 | capA | capsular polysaccharide type 5/8 biosynthesis protein CapA |
| WP_001548082.1 | -1.53 | 1.4E-04 |  | thermonuclease family protein |
| WP_000196911.1 | -1.53 | 3.7E-03 |  | ACT domain-containing protein |
| WP_000060776.1 | -1.53 | 6.8E-06 | mnhA2 | Na+/H+ antiporter Mnh2 subunit A |
| WP_000368687.1 | -1.52 | 1.4E-02 |  | hypothetical protein |
| WP_000422858.1 | -1.49 | 6.8E-06 | hxlA | 3-hexulose-6-phosphate synthase |
| WP_000635623.1 | -1.48 | 4.7E-03 | hisH | imidazole glycerol phosphate synthase subunit HisH |
| WP_000587958.1 | -1.48 | 8.0E-06 | vraX | C1q-binding complement inhibitor VraX |
| WP_000142189.1 | -1.48 | 3.4E-03 |  | acyl-CoA dehydrogenase family protein |
| WP_000277968.1 | -1.47 | 7.9E-04 | hutG | formimidoylglutamase |
| WP_000680947.1 | -1.47 | 1.8E-05 | secA2 | accessory Sec system translocase SecA2 |
| WP_000581792.1 | -1.47 | 3.4E-03 |  | heavy metal-associated domain-containing protein |
| WP_000810443.1 | -1.46 | 1.1E-03 |  | hypothetical protein |
| WP_001075826.1 | -1.46 | 3.8E-04 |  | M20 family metallopeptidase |
| WP_000129411.1 | -1.46 | 3.2E-02 | arcA | arginine deiminase |
| WP_000735558.1 | -1.46 | 4.5E-05 |  | hypothetical protein |
| WP_001044915.1 | -1.46 | 1.5E-03 |  | tyrosine-type recombinase/integrase |
| WP_000586140.1 | -1.45 | 1.0E-03 |  | hypothetical protein |
| WP_000448901.1 | -1.45 | 9.4E-04 |  | alpha/beta hydrolase |
| WP_001255791.1 | -1.45 | 1.5E-04 | ilvD | dihydroxy-acid dehydratase |
| WP_000154514.1 | -1.43 | 8.4E-03 |  | 3-hydroxyacyl-CoA dehydrogenase/enoyl-CoA hydratase family protein |
| WP_000571736.1 | -1.43 | 1.6E-02 | hisA | 1-(5-phosphoribosyl)-5-((5-phosphoribosylamino)methylideneamino)  imidazole-4-carboxamide isomerase |
| WP_000145163.1 | -1.42 | 2.8E-03 |  | sterile alpha motif-like domain-containing protein |
| WP_000163283.1 | -1.42 | 1.2E-04 |  | type 1 glutamine amidotransferase domain-containing protein |
| WP_000410771.1 | -1.42 | 1.7E-02 |  | HdeD family acid-resistance protein |
| WP_001051594.1 | -1.42 | 1.9E-03 | hxlB | 6-phospho-3-hexuloisomerase |
| WP_000763768.1 | -1.41 | 1.9E-04 |  | general stress protein |
| WP_000421701.1 | -1.40 | 6.9E-05 | betB | betaine-aldehyde dehydrogenase |
| WP_000955777.1 | -1.39 | 2.4E-04 | adhE | bifunctional acetaldehyde-CoA/alcohol dehydrogenase |
| WP_000762708.1 | -1.37 | 9.7E-05 |  | putative metal homeostasis protein |
| WP_001037807.1 | -1.37 | 1.2E-04 |  | DoxX family protein |
| WP_001795496.1 | -1.36 | 2.9E-03 | crtO | glycosyl-4,4'-diaponeurosporenoate acyltransferase |
| WP_000655692.1 | -1.36 | 2.2E-03 |  | aminotransferase class I/II-fold pyridoxal phosphate-dependent enzyme |
| WP_001013484.1 | -1.35 | 3.3E-04 |  | cation diffusion facilitator family transporter |
| WP_000581552.1 | -1.35 | 7.2E-04 |  | lantibiotic immunity ABC transporter MutE/EpiE family permease subunit |
| USA300HOU_RS10525 | -1.33 | 1.2E-03 | eap | extracellular adherence protein Eap/Map |
| WP_000827000.1 | -1.32 | 1.1E-04 |  | hypothetical protein |
| USA300HOU_RS15940 | -1.32 | 1.8E-05 | sqr | type II sulfide:quinone oxidoreductase Sqr |
| WP_000030057.1 | -1.32 | 5.5E-03 |  | aspartate aminotransferase family protein |
| WP_000072147.1 | -1.31 | 8.8E-05 | fetB | iron export ABC transporter permease subunit FetB |
| WP_000277602.1 | -1.29 | 1.8E-05 |  | universal stress protein |
| WP_001058981.1 | -1.29 | 3.7E-05 | clpL | ATP-dependent Clp protease ATP-binding subunit ClpL |
| WP_000214557.1 | -1.28 | 7.9E-04 | ilvC | ketol-acid reductoisomerase |
| WP_000158433.1 | -1.28 | 6.3E-05 | gtfA | accessory Sec system glycosyltransferase GtfA |
| WP_000950548.1 | -1.27 | 6.0E-05 | mnhD2 | Na+/H+ antiporter Mnh2 subunit D |
| WP_000690439.1 | -1.27 | 4.4E-03 | pdxT | pyridoxal 5'-phosphate synthase glutaminase subunit PdxT |
| WP_000215236.1 | -1.27 | 3.7E-05 |  | Asp23/Gls24 family envelope stress response protein |
| WP_000825819.1 | -1.26 | 7.4E-05 | amaP | alkaline shock response membrane anchor protein AmaP |
| WP_000593153.1 | -1.26 | 3.8E-05 | gtfB | accessory Sec system glycosylation chaperone GtfB |
| WP_000094576.1 | -1.25 | 9.8E-04 |  | 2-isopropylmalate synthase |
| WP_001154618.1 | -1.24 | 1.9E-02 |  | thiolase family protein |
| WP_000034728.1 | -1.23 | 2.9E-03 | pdxS | pyridoxal 5'-phosphate synthase lyase subunit PdxS |
| WP_000793009.1 | -1.23 | 3.3E-04 |  | nucleobase:cation symporter-2 family protein |
| WP_001206093.1 | -1.22 | 5.6E-04 |  | aldehyde dehydrogenase family protein |
| WP_000616642.1 | -1.22 | 2.1E-04 | mnhF2 | Na+/H+ antiporter Mnh2 subunit F |
| WP_000047820.1 | -1.22 | 2.2E-03 | ilvB | biosynthetic-type acetolactate synthase large subunit |
| WP_000702782.1 | -1.21 | 3.8E-05 | csbB | lipoteichoic acid-specific glycosylation protein CsbB |
| WP_000565302.1 | -1.21 | 1.2E-02 | cap8C | type 8 capsular polysaccharide synthesis protein Cap8C |
| WP_000183771.1 | -1.21 | 1.1E-02 |  | (S)-acetoin forming diacetyl reductase |
| WP_000175846.1 | -1.20 | 1.5E-03 |  | PLP-dependent aspartate aminotransferase family protein |
| WP_000661906.1 | -1.20 | 1.2E-03 | mnhB2 | Na+/H+ antiporter Mnh2 subunit B |
| WP_001048985.1 | -1.19 | 2.2E-03 | mnhC2 | Na+/H+ antiporter Mnh2 subunit C |
| WP_000910581.1 | -1.19 | 3.0E-03 |  | hypothetical protein |
| WP_001108126.1 | -1.19 | 1.9E-03 |  | sucrose-specific PTS transporter subunit IIBC |
| WP_000406611.1 | -1.18 | 8.0E-05 | mnhG2 | Na+/H+ antiporter Mnh2 subunit G |
| WP_000052078.1 | -1.17 | 3.2E-02 |  | hypothetical protein |
| WP_000174578.1 | -1.17 | 7.9E-04 |  | DNA-binding protein |
| WP_000136166.1 | -1.16 | 2.5E-02 | argF | ornithine carbamoyltransferase |
| WP_000769720.1 | -1.16 | 1.4E-04 |  | PepSY domain-containing protein |
| WP_001071973.1 | -1.16 | 3.7E-02 | mnhE2 | Na+/H+ antiporter Mnh2 subunit E |
| WP_000002683.1 | -1.15 | 2.5E-04 |  | DUF2273 domain-containing protein |
| WP_001024094.1 | -1.14 | 1.7E-03 |  | SDR family oxidoreductase |
| WP_000356963.1 | -1.14 | 3.3E-03 |  | hypothetical protein |
| WP_000655875.1 | -1.14 | 7.0E-03 |  | DUF5081 family protein |
| WP_000160456.1 | -1.14 | 2.1E-04 |  | NAD(P)/FAD-dependent oxidoreductase |
| WP_001060912.1 | -1.13 | 8.7E-04 |  | aldehyde reductase |
| WP_000931237.1 | -1.13 | 3.3E-05 |  | aldo/keto reductase |
| WP_000691766.1 | -1.13 | 1.1E-04 |  | RidA family protein |
| WP_001044560.1 | -1.12 | 2.7E-03 | isaB | immunodominant staphylococcal antigen IsaB |
| WP_001151900.1 | -1.12 | 3.0E-05 | sasF | cell-wall-anchored protein SasF |
| WP_000769689.1 | -1.12 | 1.3E-04 |  | MAP domain-containing protein |
| WP_000868999.1 | -1.12 | 1.3E-04 | spoVG | septation regulator SpoVG |
| WP_000549278.1 | -1.12 | 9.4E-05 | essC | type VII secretion protein EssC |
| WP_001006445.1 | -1.11 | 4.1E-03 | norC | multidrug efflux MFS transporter NorC |
| WP_000111118.1 | -1.10 | 6.5E-03 |  | FeoB-associated Cys-rich membrane protein |
| WP_001165058.1 | -1.10 | 2.4E-02 |  | protein VraC |
| WP_000974460.1 | -1.10 | 7.9E-04 |  | organic hydroperoxide resistance protein |
| WP_000221958.1 | -1.09 | 7.1E-04 | leuB | 3-isopropylmalate dehydrogenase |
| WP_001802886.1 | -1.09 | 1.1E-03 |  | YagU family protein |
| WP_000249804.1 | -1.08 | 5.8E-03 | arcA | arginine deiminase |
| WP_001240826.1 | -1.08 | 8.2E-03 | esxA | WXG100 family type VII secretion effector EsxA |
| WP_000030812.1 | -1.07 | 4.1E-03 | purM | phosphoribosylformylglycinamidine cyclo-ligase |
| WP_000247465.1 | -1.07 | 4.6E-03 |  | CHAP domain-containing protein |
| WP_001198024.1 | -1.07 | 4.3E-02 |  | TM2 domain-containing protein |
| WP_000077318.1 | -1.07 | 2.1E-03 |  | bifunctional homocysteine  S-methyltransferase/methylenetetrahydrofolate reductase |
| WP_000238664.1 | -1.07 | 6.6E-03 | purN | phosphoribosylglycinamide formyltransferase |
| WP_000239545.1 | -1.07 | 6.6E-04 | lukS-PV | Panton-Valentine bi-component leukocidin subunit S |
| WP_000686168.1 | -1.06 | 9.4E-05 |  | NAD(P)/FAD-dependent oxidoreductase |
| WP_001028431.1 | -1.06 | 2.3E-03 |  | NADPH-dependent FMN reductase |
| WP_000700921.1 | -1.06 | 4.9E-04 |  | carboxylesterase/lipase family protein |
| USA300HOU_RS00925 | -1.06 | 6.1E-03 |  | acyl-CoA/acyl-ACP dehydrogenase |
| WP_001092004.1 | -1.05 | 7.6E-04 |  | DUF2294 domain-containing protein |
| WP_000037332.1 | -1.05 | 5.4E-03 | cap8B | type 8 capsular polysaccharide synthesis protein Cap8B |
| WP_000648118.1 | -1.05 | 3.0E-04 |  | YtxH domain-containing protein |
| WP_000072278.1 | -1.04 | 7.9E-04 |  | sensor histidine kinase KdpD |
| WP_001045421.1 | -1.04 | 2.5E-02 |  | alpha/beta hydrolase |
| WP_000634175.1 | -1.04 | 8.5E-04 |  | universal stress protein |
| WP_000421410.1 | -1.03 | 5.0E-04 | xpt | xanthine phosphoribosyltransferase |
| WP_000709291.1 | -1.02 | 1.1E-02 | purH | bifunctional phosphoribosylaminoimidazolecarboxamide  formyltransferase/IMP cyclohydrolase |
| WP_001146763.1 | -1.02 | 5.6E-03 |  | carboxymuconolactone decarboxylase family protein |
| WP_001101908.1 | -1.02 | 3.4E-03 | purD | phosphoribosylamine--glycine ligase |
| WP_001052483.1 | -1.02 | 1.2E-02 |  | hypothetical protein |
| WP_000691541.1 | -1.01 | 2.7E-02 |  | S8 family serine peptidase |
| WP_000840811.1 | -1.00 | 8.6E-03 |  | DUF1641 domain-containing protein |

**Table S2: USA300 MRSA downregulated genes after exposure to *D. incerta*.** Genes with adjusted p val < 0.05 and fold-change >2 are included. Rows are sorted by fold change.

| **RefSeqID** | **Log_2_(FC)** | **adj p val** | **Gene Symbol** | **Protein** |
| --- | --- | --- | --- | --- |
| WP_001790708.1 | 2.68 | 4.2E-02 |  | hypothetical protein |
| WP_000616842.1 | 2.57 | 1.8E-04 |  | ABC transporter ATP-binding protein |
| WP_000070866.1 | 2.08 | 2.6E-06 |  | Dps family protein |
| WP_001178619.1 | 2.06 | 9.8E-05 |  | solute carrier family 23 protein |
| WP_000066062.1 | 2.04 | 4.4E-04 | argH | argininosuccinate lyase |
| WP_001789981.1 | 2.04 | 4.6E-02 |  | hypothetical protein |
| WP_000754443.1 | 1.97 | 9.5E-05 |  | siderophore ABC transporter substrate-binding protein |
| WP_000660045.1 | 1.87 | 3.6E-03 |  | argininosuccinate synthase |
| WP_000932073.1 | 1.82 | 1.6E-02 |  | ferrous iron transport protein A |
| WP_000627551.1 | 1.75 | 1.2E-02 |  | DUF3969 family protein |
| WP_000003870.1 | 1.67 | 3.3E-05 | pyrR | bifunctional pyr operon transcriptional regulator/uracil phosphoribosyltransferase PyrR |
| WP_001016166.1 | 1.63 | 8.8E-05 |  | aspartate carbamoyltransferase catalytic subunit |
| WP_001245577.1 | 1.62 | 9.7E-03 |  | iron chelate uptake ABC transporter family permease subunit |
| WP_000213873.1 | 1.61 | 1.8E-05 |  | DHA2 family efflux MFS transporter permease subunit |
| WP_000539688.1 | 1.59 | 3.1E-02 |  | DUF2951 domain-containing protein |
| WP_000793605.1 | 1.57 | 3.1E-04 | argF | ornithine carbamoyltransferase |
| WP_000738247.1 | 1.57 | 2.0E-04 |  | HlyD family efflux transporter periplasmic adaptor subunit |
| WP_000083807.1 | 1.56 | 3.7E-05 |  | PTS mannitol transporter subunit IICB |
| WP_000590809.1 | 1.54 | 1.1E-02 |  | amino acid ABC transporter ATP-binding protein |
| WP_000402904.1 | 1.53 | 3.5E-02 |  | hypothetical protein |
| WP_001040261.1 | 1.52 | 9.9E-03 |  | hypothetical protein |
| WP_000052781.1 | 1.52 | 3.1E-05 | ahpC | alkyl hydroperoxide reductase subunit C |
| WP_001074342.1 | 1.50 | 2.4E-04 | arcC | carbamate kinase |
| WP_000612128.1 | 1.49 | 2.5E-02 |  | urease subunit beta |
| WP_000432077.1 | 1.49 | 2.1E-04 |  | YfcC family protein |
| WP_000930486.1 | 1.49 | 7.4E-05 | ahpF | alkyl hydroperoxide reductase subunit F |
| WP_000649907.1 | 1.41 | 8.0E-03 |  | ABC transporter permease subunit |
| WP_000767028.1 | 1.40 | 3.0E-04 |  | dihydroorotase |
| USA300HOU_RS09550 | 1.40 | 9.2E-03 |  | hypothetical protein |
| WP_000876318.1 | 1.36 | 1.3E-02 |  | ABC transporter permease |
| WP_000828726.1 | 1.26 | 2.3E-03 |  | sodium-dependent transporter |
| WP_000549734.1 | 1.24 | 1.7E-02 | cidA | holin-like murein hydrolase modulator CidA |
| WP_000798979.1 | 1.23 | 8.7E-04 |  | PepSY domain-containing protein |
| WP_001549577.1 | 1.21 | 3.4E-03 |  | YjiH family protein |
| WP_000571584.1 | 1.18 | 8.5E-04 | gltS | sodium/glutamate symporter |
| WP_000064778.1 | 1.15 | 1.2E-04 |  | amino acid permease |
| WP_001200541.1 | 1.11 | 1.6E-02 | crcB | fluoride efflux transporter CrcB |
| WP_000181819.1 | 1.11 | 3.0E-02 |  | dUTP pyrophosphatase |
| WP_000210828.1 | 1.11 | 1.0E-03 | tdcB | bifunctional threonine ammonia-lyase/L-serine ammonia-lyase TdcB |
| WP_000755150.1 | 1.10 | 8.4E-03 |  | head-tail adaptor protein |
| WP_000985235.1 | 1.10 | 1.3E-03 |  | phage/plasmid primase, P4 family |
| WP_000447733.1 | 1.09 | 1.1E-04 | xerD | site-specific tyrosine recombinase XerD |
| WP_011447039.1 | 1.09 | 8.9E-04 |  | putative holin-like toxin |
| WP_000725225.1 | 1.08 | 1.2E-04 |  | CHAP domain-containing protein |
| WP_000414205.1 | 1.08 | 4.8E-03 |  | hypothetical protein |
| WP_001008722.1 | 1.08 | 3.8E-04 | uhpT | hexose-6-phosphate:phosphate antiporter |
| WP_001120199.1 | 1.06 | 3.5E-02 |  | DUF771 domain-containing protein |
| WP_001795631.1 | 1.05 | 2.5E-04 | tatC | twin-arginine translocase subunit TatC |
| WP_000789821.1 | 1.05 | 4.4E-02 | isdC | heme uptake protein IsdC |
| WP_000959424.1 | 1.05 | 2.2E-03 | ald | alanine dehydrogenase |
| WP_000861038.1 | 1.05 | 7.4E-03 |  | CHAP domain-containing protein |
| WP_000414685.1 | 1.04 | 1.3E-04 | norB | multidrug efflux MFS transporter NorB |
| WP_001549178.1 | 1.04 | 4.5E-02 |  | DUF2482 family protein |
| WP_001261987.1 | 1.00 | 1.9E-03 | rimM | ribosome maturation factor RimM |

**Table S3: USA300 MRSA upregulated genes after exposure to *D. incerta*.** Genes with adjusted p val < 0.05 and fold-change >2 are included. Rows are sorted by fold change.

| **RefSeqID** | **log_2_(FC)** | **adj. p val** | **Gene Symbol** | **Protein** |
| --- | --- | --- | --- | --- |
| WP_000129411.1 | -3.94 | 5.15E-09 | arcA | arginine deiminase |
| WP_000136166.1 | -3.61 | 1.07E-07 | argF | ornithine carbamoyltransferase |
| WP_001005842.1 | -3.14 | 1.07E-07 | arcD | arginine-ornithine antiporter |
| WP_000669519.1 | -2.53 | 1.71E-05 | spn | myeloperoxidase inhibitor SPIN |
| WP_000660034.1 | -2.50 | 4.04E-07 | arcC | carbamate kinase |
| WP_000793009.1 | -2.04 | 1.07E-07 |  | nucleobase:cation symporter-2 family protein |
| WP_001005511.1 | -1.95 | 5.32E-07 |  | fructosamine kinase family protein |
| WP_000421410.1 | -1.92 | 1.07E-07 | xpt | xanthine phosphoribosyltransferase |
| WP_001108126.1 | -1.92 | 4.17E-06 |  | sucrose-specific PTS transporter subunit IIBC |
| WP_000410771.1 | -1.83 | 1.50E-06 |  | HdeD family acid-resistance protein |
| WP_000138214.1 | -1.83 | 5.66E-06 |  | Crp/Fnr family transcriptional regulator |
| WP_000154514.1 | -1.78 | 2.14E-06 |  | 3-hydroxyacyl-CoA dehydrogenase/enoyl-CoA hydratase family protein |
| WP_000142189.1 | -1.77 | 8.13E-07 |  | acyl-CoA dehydrogenase family protein |
| WP_001154618.1 | -1.75 | 4.59E-05 |  | thiolase family protein |
| WP_001056223.1 | -1.68 | 4.18E-07 | clfA | MSCRAMM family adhesin clumping factor ClfA |
| WP_001549197.1 | -1.66 | 8.70E-05 |  | delta-lysin family phenol-soluble modulin |
| WP_001008401.1 | -1.56 | 1.56E-06 |  | class I adenylate-forming enzyme family protein |
| WP_000066521.1 | -1.56 | 1.12E-06 | betA | choline dehydrogenase |
| WP_000238664.1 | -1.53 | 2.06E-05 | purN | phosphoribosylglycinamide formyltransferase |
| WP_001075826.1 | -1.49 | 1.56E-06 |  | M20 family metallopeptidase |
| WP_000565302.1 | -1.48 | 1.13E-06 | cap8C | type 8 capsular polysaccharide synthesis protein Cap8C |
| WP_001549607.1 | -1.48 | 4.18E-07 |  | MAP domain-containing protein |
| WP_000483713.1 | -1.46 | 5.94E-06 | purF | amidophosphoribosyltransferase |
| WP_000264071.1 | -1.44 | 8.43E-07 | guaB | IMP dehydrogenase |
| WP_000037332.1 | -1.43 | 1.32E-06 | cap8B | type 8 capsular polysaccharide synthesis protein Cap8B |
| WP_000030812.1 | -1.43 | 4.17E-06 | purM | phosphoribosylformylglycinamidine cyclo-ligase |
| WP_000424966.1 | -1.43 | 1.07E-07 | guaA | glutamine-hydrolyzing GMP synthase |
| WP_001548082.1 | -1.43 | 0.00014035 |  | thermonuclease family protein |
| WP_000448901.1 | -1.38 | 3.44E-06 |  | alpha/beta hydrolase |
| WP_001006445.1 | -1.37 | 5.14E-06 | norC | multidrug efflux MFS transporter NorC |
| WP_000857483.1 | -1.36 | 5.94E-06 | hyl | alpha-hemolysin |
| WP_000730043.1 | -1.33 | 1.01E-05 |  | SDR family oxidoreductase |
| WP_000998767.1 | -1.33 | 8.13E-07 | hutI | imidazolonepropionase |
| WP_000459057.1 | -1.30 | 4.69E-05 | cap8E | type 8 capsular polysaccharide synthesis protein Cap8E |
| WP_000709291.1 | -1.29 | 2.40E-05 | purH | bifunctional phosphoribosylaminoimidazolecarboxamide  formyltransferase/IMP cyclohydrolase |
| WP_000032727.1 | -1.28 | 2.52E-07 | purL | phosphoribosylformylglycinamidine synthase subunit PurL |
| USA300HOU_RS00815 | -1.27 | 2.11E-05 | cap8D | type 8 capsular polysaccharide synthesis protein Cap8D |
| WP_000595324.1 | -1.27 | 0.000190134 | lukG | bi-component leukocidin LukGH subunit G |
| WP_000601724.1 | -1.27 | 3.36E-05 | nrdG | anaerobic ribonucleoside-triphosphate reductase activating protein |
| WP_000160456.1 | -1.26 | 1.89E-06 |  | NAD(P)/FAD-dependent oxidoreductase |
| WP_000955777.1 | -1.26 | 8.72E-06 | adhE | bifunctional acetaldehyde-CoA/alcohol dehydrogenase |
| WP_000791407.1 | -1.25 | 0.000632883 | lukH | bi-component leukocidin LukGH subunit H |
| WP_001101908.1 | -1.24 | 4.99E-05 | purD | phosphoribosylamine--glycine ligase |
| WP_001226823.1 | -1.24 | 1.89E-06 | hutU | urocanate hydratase |
| WP_001802886.1 | -1.23 | 3.04E-06 |  | YagU family protein |
| WP_001177186.1 | -1.23 | 0.000145566 |  | hypothetical protein |
| WP_001790533.1 | -1.23 | 0.005305845 |  | hypothetical protein |
| WP_000676548.1 | -1.21 | 8.72E-06 | sspA | Glu-specific serine endopeptidase SspA |
| WP_001789452.1 | -1.19 | 9.49E-07 |  | acyl CoA:acetate/3-ketoacid CoA transferase |
| WP_000680947.1 | -1.18 | 8.39E-06 | secA2 | accessory Sec system translocase SecA2 |
| WP_001028293.1 | -1.18 | 0.000115679 | cap8F | type 8 capsular polysaccharide synthesis protein Cap8F |
| WP_000072147.1 | -1.18 | 8.72E-06 | fetB | iron export ABC transporter permease subunit FetB |
| WP_000446299.1 | -1.17 | 3.23E-05 | capA | capsular polysaccharide type 5/8 biosynthesis protein CapA |
| WP_000517908.1 | -1.16 | 0.000355507 | rpmB | 50S ribosomal protein L28 |
| WP_000002683.1 | -1.16 | 1.05E-06 |  | DUF2273 domain-containing protein |
| WP_000421701.1 | -1.15 | 9.63E-07 | betB | betaine-aldehyde dehydrogenase |
| USA300HOU_RS00925 | -1.15 | 0.000498107 |  | acyl-CoA/acyl-ACP dehydrogenase |
| WP_001822934.1 | -1.14 | 0.015404406 |  | MAP domain-containing protein |
| WP_000071901.1 | -1.14 | 1.12E-06 |  | pyridoxamine 5'-phosphate oxidase family protein |
| WP_000158433.1 | -1.13 | 2.58E-06 | gtfA | accessory Sec system glycosyltransferase GtfA |
| WP_001096791.1 | -1.13 | 0.000180667 |  | lantibiotic protection ABC transporter ATP-binding subunit |
| USA300HOU_RS16045 | -1.13 | 5.87E-05 | hisF | imidazole glycerol phosphate synthase subunit HisF |
| WP_000634105.1 | -1.12 | 0.000387105 |  | cysteine synthase family protein |
| WP_001071717.1 | -1.10 | 1.76E-05 | nrdD | anaerobic ribonucleoside-triphosphate reductase |
| WP_000699538.1 | -1.09 | 0.001705888 |  | PTS sugar transporter subunit IIB |
| WP_000827000.1 | -1.08 | 3.09E-05 |  | hypothetical protein |
| WP_000593153.1 | -1.07 | 8.44E-06 | gtfB | accessory Sec system glycosylation chaperone GtfB |
| WP_000825819.1 | -1.06 | 1.89E-06 | amaP | alkaline shock response membrane anchor protein AmaP |
| WP_001216898.1 | -1.06 | 0.001350064 |  | CatB-related O-acetyltransferase |
| WP_000587958.1 | -1.05 | 0.000650853 | vraX | C1q-binding complement inhibitor VraX |
| WP_000163988.1 | -1.05 | 1.66E-05 |  | 6-phospho-beta-glucosidase |
| WP_000711870.1 | -1.05 | 8.92E-05 |  | BCCT family transporter |
| USA300HOU_RS00830 | -1.05 | 0.000712511 | cap8G | type 8 capsular polysaccharide synthesis protein Cap8G |
| WP_000581552.1 | -1.04 | 2.66E-06 |  | lantibiotic immunity ABC transporter MutE/EpiE family permease subunit |
| WP_000661906.1 | -1.03 | 0.000387105 | mnhB2 | Na+/H+ antiporter Mnh2 subunit B |
| WP_000848350.1 | -1.03 | 0.000650687 | purS | phosphoribosylformylglycinamidine synthase subunit PurS |
| WP_000634175.1 | -1.03 | 0.000247258 |  | universal stress protein |
| WP_000277968.1 | -1.03 | 5.38E-05 | hutG | formimidoylglutamase |
| WP_000943832.1 | -1.02 | 1.50E-06 | lip2 | YSIRK domain-containing triacylglycerol lipase Lip2/Geh |
| WP_000571736.1 | -1.02 | 0.012496071 | hisA | 1-(5-phosphoribosyl)-5-((5-phosphoribosylamino)methylideneamino) imidazole-4-carboxamide isomerase |
| WP_000239610.1 | -1.02 | 0.005137035 |  | phospholipase |
| WP_001077722.1 | -1.01 | 7.85E-05 |  | YjiH family protein |
| WP_000066937.1 | -1.01 | 0.001400361 |  | glucose PTS transporter subunit IIA |
| WP_000265043.1 | -1.01 | 0.049972722 |  | DUF1514 family protein |

**Table S4: *S. aureus SA113* downregulated genes after exposure to *D. incerta*.** Genes with adjusted p val < 0.05 and fold-change >2 are included. Rows are sorted by fold change.

| **RefSeqID** | **Log_2_(FC)** | **adj p val** | **Gene Symbol** | **Protein** |
| --- | --- | --- | --- | --- |
| WP_000066062.1 | 3.91 | 6.20E-09 | argH | argininosuccinate lyase |
| WP_000660045.1 | 3.87 | 1.89E-06 |  | argininosuccinate synthase |
| WP_000649907.1 | 3.37 | 1.13E-06 |  | ABC transporter permease subunit |
| WP_001549577.1 | 3.28 | 6.20E-09 |  | YjiH family protein |
| WP_001040261.1 | 3.27 | 0.011047429 |  | hypothetical protein |
| WP_000590809.1 | 3.08 | 1.07E-05 |  | amino acid ABC transporter ATP-binding protein |
| WP_000616842.1 | 2.49 | 0.000117104 |  | ABC transporter ATP-binding protein |
| WP_000138932.1 | 2.36 | 0.040326939 | csoR | copper-sensing transcriptional repressor CsoR |
| WP_000410720.1 | 2.24 | 0.000881333 |  | hypothetical protein |
| WP_000499647.1 | 1.92 | 0.003000638 |  | DNA damage-induced cell division inhibitor SosA |
| WP_000404422.1 | 1.90 | 0.004719098 |  | L-threonylcarbamoyladenylate synthase |
| WP_000754443.1 | 1.88 | 3.88E-05 |  | siderophore ABC transporter substrate-binding protein |
| WP_000619366.1 | 1.75 | 0.005118832 |  | LeuA family protein |
| WP_000876318.1 | 1.70 | 1.60E-05 |  | ABC transporter permease |
| WP_000539688.1 | 1.70 | 0.008718631 |  | DUF2951 domain-containing protein |
| WP_000627551.1 | 1.58 | 0.000282396 |  | DUF3969 family protein |
| WP_000932073.1 | 1.53 | 0.002774626 |  | ferrous iron transport protein A |
| WP_000593436.1 | 1.41 | 0.000226181 |  | ABC transporter ATP-binding protein |
| WP_001245577.1 | 1.40 | 0.004008149 |  | iron chelate uptake ABC transporter family permease subunit |
| WP_000472235.1 | 1.38 | 1.50E-06 |  | ABC transporter permease |
| WP_000567394.1 | 1.38 | 0.004900273 |  | phage tail family protein |
| WP_001548981.1 | 1.32 | 0.041244033 | sel26 | staphylococcal enterotoxin type 26 |
| WP_000584765.1 | 1.32 | 1.15E-05 |  | ABC transporter permease |
| WP_000414205.1 | 1.31 | 9.14E-05 |  | hypothetical protein |
| WP_000244278.1 | 1.30 | 0.004206723 |  | GNAT family N-acetyltransferase |
| WP_001246698.1 | 1.29 | 0.005609459 | isdD | iron-regulated surface determinant protein IsdD |
| WP_000476873.1 | 1.29 | 0.019752112 |  | DUF1672 domain-containing protein |
| WP_000185864.1 | 1.28 | 0.017916987 |  | polysaccharide biosynthesis tyrosine autokinase |
| WP_000283016.1 | 1.28 | 8.70E-05 |  | Y-family DNA polymerase |
| WP_000173869.1 | 1.24 | 4.74E-05 |  | ABC transporter ATP-binding protein |
| WP_000200684.1 | 1.24 | 0.00014035 |  | PTS transporter subunit IIC |
| WP_000210828.1 | 1.22 | 1.31E-05 | tdcB | bifunctional threonine ammonia-lyase/L-serine ammonia-lyase TdcB |
| WP_000876201.1 | 1.21 | 0.045884893 | msaC | sarA expression modulator MsaC |
| WP_000064778.1 | 1.20 | 4.91E-06 |  | amino acid permease |
| WP_000070866.1 | 1.20 | 0.000244369 |  | Dps family protein |
| WP_001217917.1 | 1.19 | 0.019097999 | bioD | dethiobiotin synthase |
| USA300HOU_RS09550 | 1.19 | 0.002268714 |  | hypothetical protein |
| WP_001095260.1 | 1.19 | 0.000275788 |  | Fur family transcriptional regulator |
| WP_000166917.1 | 1.19 | 0.008823963 |  | M23 family metallopeptidase |
| WP_001229077.1 | 1.19 | 1.56E-06 | cntA | staphylopine-dependent metal ABC  transporter substrate-binding protein CntA |
| WP_001105087.1 | 1.18 | 0.044795892 | cstR | persulfide-sensing transcriptional repressor CstR |
| WP_000725225.1 | 1.18 | 2.00E-05 |  | CHAP domain-containing protein |
| WP_000727762.1 | 1.18 | 2.70E-05 | adcA | zinc ABC transporter substrate-binding lipoprotein AdcA |
| WP_000623471.1 | 1.16 | 0.000347763 |  | CrcB family protein |
| WP_001789875.1 | 1.16 | 0.002145844 | prli42 | stressosome-associated protein Prli42 |
| WP_000706137.1 | 1.15 | 6.61E-05 |  | NAD-dependent formate dehydrogenase |
| WP_000934424.1 | 1.14 | 7.95E-05 | sdrD | MSCRAMM family adhesin SdrD |
| WP_000534425.1 | 1.12 | 1.14E-05 |  | amino acid permease |
| WP_001789408.1 | 1.12 | 0.03075173 |  | NAD-dependent epimerase/dehydratase family protein |
| WP_000213873.1 | 1.10 | 4.26E-07 |  | DHA2 family efflux MFS transporter permease subunit |
| WP_000738247.1 | 1.10 | 6.10E-06 |  | HlyD family efflux transporter periplasmic adaptor subunit |
| WP_000728761.1 | 1.10 | 6.00E-06 | spa | staphylococcal protein A |
| WP_000335332.1 | 1.10 | 0.033826253 |  | MepB family protein |
| WP_001802312.1 | 1.09 | 0.014424659 |  | hypothetical protein |
| WP_000870806.1 | 1.09 | 0.019097999 |  | bacteriocin-associated integral membrane family protein |
| WP_000083807.1 | 1.07 | 5.39E-05 |  | PTS mannitol transporter subunit IICB |
| WP_000959424.1 | 1.07 | 4.17E-06 | ald | alanine dehydrogenase |
| WP_000041896.1 | 1.07 | 0.000106678 |  | superantigen-like protein SSL12 |
| WP_000876756.1 | 1.04 | 0.000155376 | sarS | HTH-type transcriptional regulator SarS |
| WP_000570808.1 | 1.04 | 0.028822046 | sbnA | 2,3-diaminopropionate biosynthesis protein SbnA |
| WP_001041662.1 | 1.04 | 0.004057948 |  | LysM peptidoglycan-binding domain-containing protein |
| WP_001220199.1 | 1.03 | 0.007204009 | isdE | heme ABC transporter substrate-binding protein IsdE |
| WP_001795631.1 | 1.01 | 9.78E-06 | tatC | twin-arginine translocase subunit TatC |
| WP_000011823.1 | 1.00 | 2.53E-06 |  | hypothetical protein |

**Table S5: *S. aureus SA113* upregulated genes after exposure to *D. incerta*.** Genes with adjusted p val < 0.05 and fold-change >2 are included. Rows are sorted by fold change.


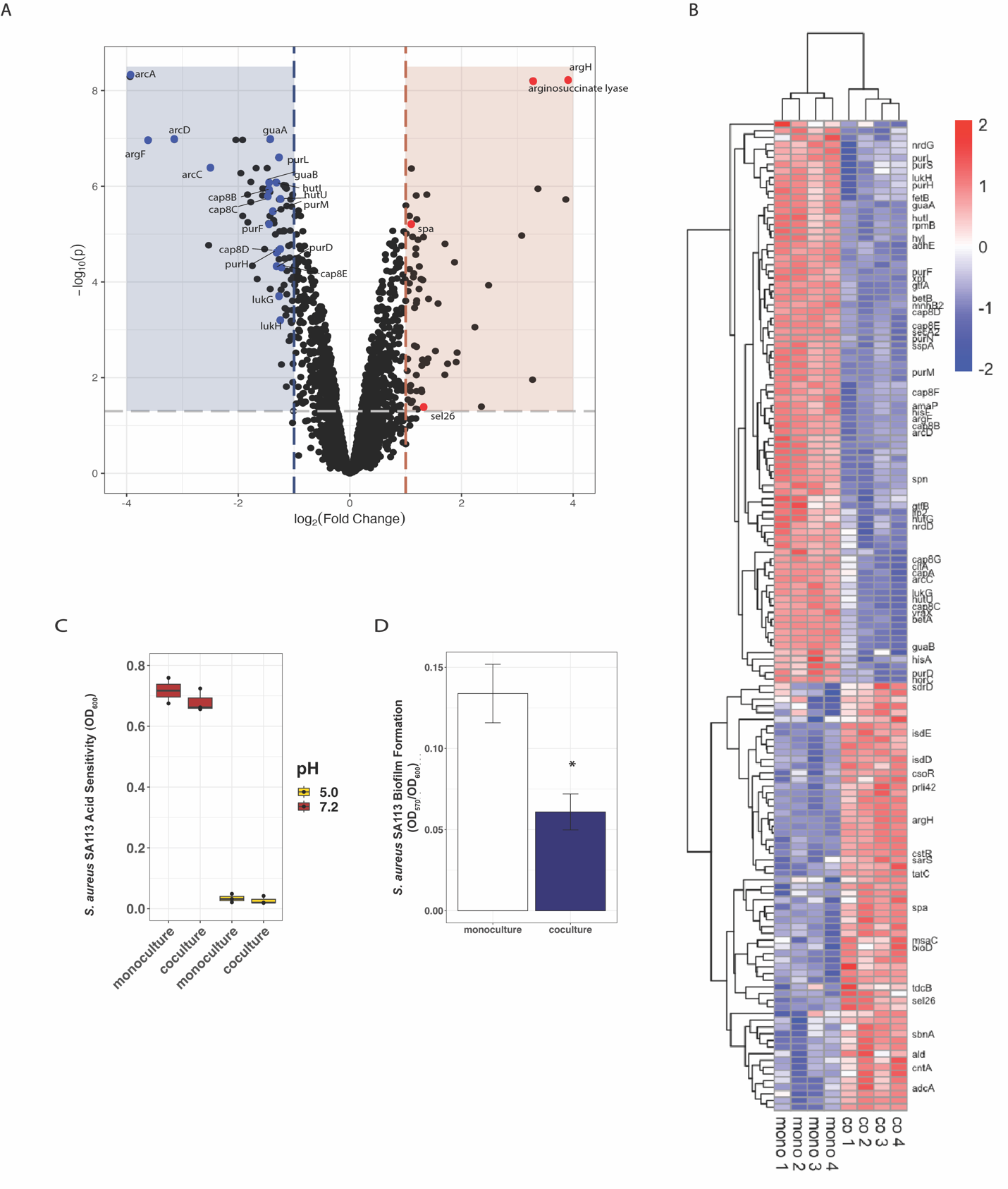


**Figure S2: Transcriptional profiling of *S. aureus* SA113 reveals similar changes in gene expression and biofilm formation compared to USA300 MRSA A)** Volcano plot of genes in *S. aureus* SA113 that were differentially expressed during *D. incerta* coculture compared to monoculture. Shaded areas highlight genes whose adjusted p < 0.05 and fold change > 2.0 (blue – downregulated, red-upregulated). Bold points mark genes of interest, which were found within operons containing multiple differentially expressed genes. **B)** Heatmap depicting differentially expressed genes in USA300 MRSA with adjusted p< 0.05 and fold change >2.0. For visualization purposes, genes were ordered by hierarchical clustering based on Pearson correlation. Values were scaled to the row mean. **C)** *S. aureus* SA 113 cell density in response to acidified media (pH = 5.0, yellow) compared to neutral pH media (red) and coculture with *D. incerta*. 3 wells per condition were used.  **D)** *S. aureus* SA113 biofilm formation measured via crystal violet stain retention (OD_570_) normalized to cell growth (OD_600_). 4 wells per condition were used.

T-test, * p < 0.05


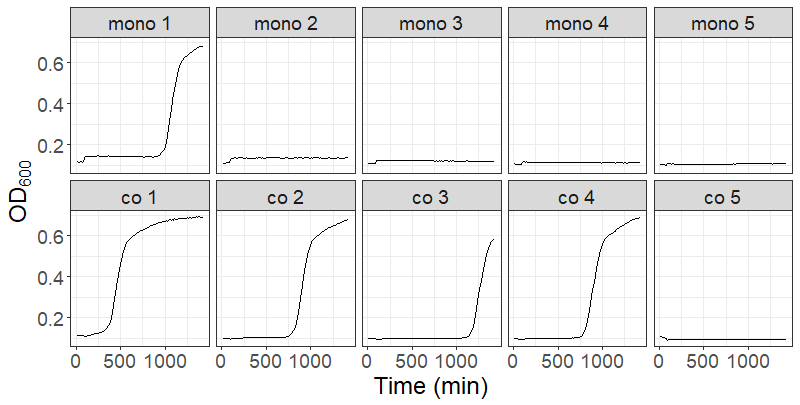


**Figure S3: Coculture with *D. incerta* increases USA300 MRSA resistance to hydrogen peroxide.** Growth curve as measure by OD_600_ over time for USA300 *S. aureus* that had been cultured alone (“mono”) or co-cultured with *D. incera* (“co”), then challenged with hydrogen peroxide. 24 hours after cocultured with *D. incerta*, USA300 MRSA cultures were resuspended to OD_600_=0.01 and allowed to grow in 96 well plates in a pre-warmed microplate reader. Hydrogen peroxide was added at a final concentration of 0.8mM at t=90 min.
